# Comparative transcriptomics and scarless genome editing uncover a periplasmic *c*-type cytochrome important for extracellular electron uptake in *Thioclava electrotropha* ElOx9^T^

**DOI:** 10.1128/aem.00250-26

**Published:** 2026-07-28

**Authors:** Joshua D. Sackett, Leah R. Trutschel, Andrew W. Grenfell, Jeffrey A. Gralnick, Annette R. Rowe

**Affiliations:** 1 Department of Microbiology, Genetics, and Immunology, Michigan State University 3078, East Lansing, Michigan, USA; 2 Department of Biological Sciences, University of Cincinnati 118729, Cincinnati, Ohio, USA; 3 BioTechnology Institute, University of Minnesota – Twin Cities 25793, St. Paul, Minnesota, USA; 4 Department of Biochemistry, Molecular Biology, and Biophysics, University of Minnesota – Twin Cities, St. Paul, Minnesota, USA; 5 Department of Biology Teaching and Learning, University of Minnesota – Twin Cities, St. Paul, Minnesota, USA; 6 Department of Plant and Microbial Biology, University of Minnesota – Twin Cities, St. Paul, Minnesota, USA; 7 Department of Earth and Environmental Sciences, Michigan State University 3078, East Lansing, Michigan, USA; Washington University in St Louis, St. Louis, Missouri, USA

**Keywords:** electrotrophy, chemolithotrophy, chemolithoautotrophy, marine sediment bacterium, gene expression, monoheme cytochrome, cathode oxidation, sulfur oxidation

## Abstract

Extracellular electron uptake (EEU) is a form of extracellular electron transfer that enables microorganisms to use solid-phase electron donors for respiration and biosynthesis, with important implications for biogeochemical cycling and biotechnology. *Thioclava electrotropha* is a metabolically versatile marine *Alphaproteobacterium* capable of autotrophic sulfur oxidation, heterotrophic growth, in addition to cathodic electron uptake despite lacking homologs to known extracellular electron transfer proteins from mineral-reducing or mineral-oxidizing microbes. Here, we explore the genetic basis for EEU in *T. electrotropha* through integrated transcriptomic, genetic, and electrochemical approaches. RNA sequencing revealed distinct transcriptional profiles for cathode oxidation versus sulfur oxidation, with 584 genes uniquely upregulated during EEU. Among the most highly upregulated genes were putative *c*-type cytochromes predicted to localize to the periplasm. A putative monoheme *c*-type cytochrome gene (AKL02_08760) designated *pmcA* showed >100-fold upregulation during cathode oxidation. We developed a genetic system enabling scarless deletions in *T. electrotropha* and demonstrated that deletion of *pmcA* significantly decreased EEU capacity, while complementation restored and enhanced electron uptake beyond wild-type levels. Phylogenetic analysis revealed PmcA homologs in 2,587 bacterial species across multiple phyla, with distribution patterns indicative of horizontal gene transfer. The *pmcA* gene belongs to a putative four-gene cytochrome *c*-multicopper oxidase operon, of which all members were upregulated during EEU, with the operon displaying atypical codon usage and elevated GC content consistent with horizontal acquisition. These findings establish a novel and potentially horizontally transferred mechanism for bacterial EEU. Further elucidation of this pathway will provide new targets for engineering enhanced bioelectrochemical systems with EEU capabilities.

**IMPORTANCE:**

Though extracellular electron transfer (EET) has been shown to drive critical biogeochemical processes in a range of environments, there are only a limited number of biomarkers that help us assign the genetic potential for EET to other microbes. This is especially true for organisms that use EET to acquire electrons from external electron donors. This work identifies a novel gene involved in the extracellular electron uptake mechanism employed by the marine sediment chemolithoautotroph and sulfur-oxidizing bacterium, *Thioclava electrotropha*. Homologs of this novel gene are found in over 2,500 species spanning multiple phyla. Though biochemical characterization is necessary to fully understand the role of this protein in EET, this work supports the potential for a widely distributed and previously uncharacterized mechanism of extracellular electron uptake. As EET has enabled the potential for multiple biotechnological applications, including microbial fuel cells and microbial electrosynthesis, further characterization of this system has implications for improved engineering of bioelectrochemical systems or use of novel electrotrophic microorganisms.

## INTRODUCTION

Extracellular electron uptake (EEU) describes the process by which some microorganisms accept electrons for respiration or biosynthesis from external sources, such as conductive minerals, other microorganisms, or in applied systems from cathodes (electrodes poised at electron-donating potentials) (1, 2). This process has important implications for understanding microbial ecology in environments where organic electron donors are limited, such as subsurface sediments. In marine sediments specifically, extracellular electron transfer processes are critical for elemental cycling where electron transfer between microorganisms and mineral phases drives mobilization of energy sources and nutrients that can influence global nutrient cycles and carbon sequestration. Further, electro(auto)trophic organisms hold considerable promise for biotechnological applications, including microbial electrosynthesis, bioremediation, biofuel production, and sustainable manufacturing processes (3–5).

Some of the best-characterized EEU pathways share homology to the MtrCAB porin-cytochrome complex in *Shewanella oneidensis* MR-1 that catalyzes iron reduction, in which MtrA and MtrC are decaheme *c*-type cytochromes and MtrB is an outer membrane ß-barrel protein that enables electron transfer across the outer membrane (6). Genetic evidence from *Rhodopseudomonas palustris* TIE-1 indicates that PioAB, which shares homology with MtrAB, is essential for iron oxidation during photoferrotrophy (7–9). Furthermore, MtoAB, another MtrAB homolog, has been implicated in Fe(II) oxidation in *S. lithotrophicus* ES-1. Purified MtoA was shown to oxidize soluble Fe(II) *in vitro* (10), and proteomic analyses demonstrated that MtoAB is specifically expressed during growth on solid Fe(II) minerals, including smectite and magnetite, but not on Fe(II)-citrate (11–13). The MtoAB pathway is thought to be preferentially used for the oxidation of solid, reduced forms of iron compared with soluble Fe^2+^, which is favored by the Cyc2 pathway characterized in the acidophile *Acidithiobacillus ferrooxidans* and demonstrated in ES-1 (11, 14, 15). A homolog of Cyc2 is the predominant outer-membrane monoheme *c*-type cytochrome in the marine neutrophilic iron oxidizer *Mariprofundus ferrooxydans* PV-1 (15, 16). While these organisms may oxidize soluble iron, the resulting insoluble product is often coordinated into remarkable mineral forms, such as the twisted ribbon stalks produced by PV-1 (17). As these interactions center on iron, there is a gap in knowledge for EEU mechanisms in other chemolitho(auto)trophs, such as sulfur oxidizers.

*Thioclava electrotropha* is a member of the *Alphaproteobacteria* family *Rhodobacteraceae*, originally isolated from marine sediments from Catalina Harbor, California, following enrichment on electrodes poised at cathodic potentials (–400 mV vs Ag/AgCl) (18). This organism exhibits remarkable metabolic versatility, capable of both chemolithoautotrophic growth using reduced sulfur compounds or hydrogen, and chemoorganotrophy utilizing a variety of organic carbon sources coupled to oxygen or nitrate reduction (19, 20). *T. electrotropha* is also capable of oxidizing a cathode poised at an environmentally and physiologically relevant electron-donating potential (18, 19, 21). Notably, this organism engages in interfacial extracellular electron transfer through an unknown mechanism that is independent of hydrogen or formate use (21). Though the genome lacks homologs to known interfacial extracellular electron transfer pathway proteins, it contains 39 genes encoding proteins with canonical “CXXCH” *c*-type heme-binding motifs (20). This enrichment in putative *c*-type cytochromes has been observed broadly in electrochemically active microbes (22, 23). Recently, a high-throughput transposon mutagenesis screen identified 54 genes required for growth utilizing a cathode as the sole electron donor in *T. electrotropha* (20). None of these 54 genes encode proteins with canonical *c*-type heme-binding motifs. Given the central role of *c*-type cytochromes in characterized EET pathways, this result raises the question whether the organism contains redundant systems for EEU or whether *T. electrotropha* utilizes a cytochrome-independent mechanism for EEU. To address this question, we used transcriptomics to identify differentially expressed genes during cathode oxidation, reasoning that genes involved in EEU would be upregulated under cathodic conditions.

Here, we present a transcriptomic, genetic, and electrochemical investigation into the extracellular electron uptake pathway employed by *Thioclava electrotropha*. This work presents evidence for a novel pathway of extracellular electron uptake that includes a previously uncharacterized putatively periplasmic monoheme *c-*type cytochrome gene (AKL02_08760), designated *pmcA*. These results provide new insights into the diversity of strategies employed by electroactive microorganisms and advances our understanding of the genetic determinants for microbe-electrode interactions.

## MATERIALS AND METHODS

### Bacterial strains and culture conditions

All bacterial strains utilized in this work are listed in Table 1. All strains were stored in 40% v/v glycerol at −80°C, unless otherwise stated. Prior to each experiment, *Thioclava electrotropha* ElOx9^T^ strains were grown on lysogeny broth plus salt and ions (LBS + Ions) agar plates and incubated at 30°C (25 g/L LB [Fisher Bioreagents], 10 g/L NaCl, 3 g/L MgCl_2_*6H_2_O, 0.15 g/L CaCl_2_*2H_2_O, with 15 g/L agar added for solid media). Single colonies were used to inoculate LBS + Ions broth or artificial low-sulfate saltwater base (SWB) (18) amended with 5 mM sodium acetate (200 RPM). For chemolithoautotrophic growth with reduced sulfur species as the electron donors, SWB media were amended with anaerobic polysulfide stock to a final concentration of 22.5 mM and allowed to oxidize overnight in a shaking incubator (200 RPM). The anaerobic polysulfide stock was prepared, as previously described (24). Abiotic oxidation of polysulfide at near-neutral pH produces primarily thiosulfate (S_2_O_3_^2−^) and elemental sulfur (S^0^), the latter of which precipitates as a white particulate suspension (25). Both S_2_O_3_^2−^ and S^0^ served as potential electron donors in chemolithoautotrophic growth experiments. The pH of the media was adjusted to 7.2 by adding sterile 1 mM phosphate buffer. *Escherichia coli* strain WM3064, a diaminopimelic acid (DAP) auxotroph, was used as the donor strain in conjugation experiments. WM3064 was grown in lysogeny broth (LB) supplemented with 300 µM DAP. Kanamycin (50 mg/L) was added to media to maintain plasmids in *T. electrotropha* and *E. coli* WM3064 or to select for transconjugants. Bioelectrochemical experiments were carried out in SWB cathode medium comprised of artificial low-sulfate saltwater base lacking organic carbon additions, vitamins, and minerals (18). Unless otherwise specified, all cultures were incubated at 30°C.

**TABLE 1:** Bacterial strains and plasmids used in this study

Strain or plasmid	Description or genotype	Source or reference
*Thioclava electrotropha* ElOx9^T^ strains		
ARR065	Electrochemically enriched and subsequently isolated from coastal sediments (Catalina Island, CA), wild type	(18, 19)
ARR288	∆*soxCD*	This study
ARR322	∆*pmcA*	This study
ARR324	∆14455	This study
ARR333	ARR065 with pBBR1MCS-2 empty vector	This study
ARR334	ARR322 with p*pmcA*^+^	This study
*E. coli* strain		
WM3064	Donor strain for conjugation; *thrB1004 pro thi rpsL hsdS lacZ∆M15 RP4-1360 ∆(araBAD)567 ∆dapA1341::[erm pir]*	W. Metcalf, University of Illinois, Urbana
Plasmids/vectors		
pSMV3	9.5-kb mobilizable suicide vector; *oriR6K, sacB,* Km^r^	(26)
pSMV3∆*soxCD*	2-kb fusion PCR fragment containing ∆*soxCD* cloned into the *Sac*I and *Bam*HI sites of pSMV3; used to make the ARR288 ∆*soxCD* strain	This study
pSMV3∆*pmcA*	2-kb fusion PCR fragment containing ∆*pmcA* cloned into the *Sac*I and *Bam*HI sites of pSMV3; used to make the ARR322 ∆*pmcA* strain	This study
pSMV3∆14455	2-kb fusion PCR fragment containing ∆14455 cloned into the *Sac*I and *Bam*HI sites of pSMV3; used to make the ARR324 ∆14455 strain	This study
pBBR1MCS-2	5.1-kb broad-host range plasmid: Km^r^, *lacZ*	(27)
p*pmcA*^+^	*pmcA* PCR fragment cloned into the *Bam*HI and *Sac*I sites of pBBR1MCS-2	This study

### Transcriptomics experimental design

We evaluated the transcriptional profiles of *Thioclava electrotropha* ElOx9^T^ under four growth conditions: aerobic heterotrophic growth, chemolithotrophic growth with reduced sulfur species, early electron uptake (24 h), and sustained electron uptake conditions (~6 days).

For aerobic heterotrophic growth, cells were grown in LBS + Ions broth overnight in triplicate (~18 h). Then, 500 μL of each replicate culture was combined with 1,000 μL of RNAProtect Bacteria Reagent (Qiagen) in a 1.5-mL tube, the tube was vortexed for 5 s, incubated at room temperature for 5 min, and cells were pelleted via centrifugation (10,000 × *g,* 10 min). The supernatant was decanted, and cell pellets were immediately frozen at −80°C until RNA purification.

For chemolithoautotrophic growth, cells were grown overnight in LBS + Ions, pelleted via centrifugation (8,000 × *g*, 5 min), and resuspended in triplicate flasks containing SWB medium with oxidized polysulfide to a final OD_600_ = 0.2. The pH of flasks was monitored hourly to prevent cell death due to acidification resulting from complete S_2_O_3_^2−^ or S^0^ oxidation. Once flask pH reached ~5.0 (approximately 4 h), 500 μL of each replicate culture was collected and processed with RNAProtect Bacteria Reagent as above. Cell pellets were immediately frozen at −80°C until RNA purification.

For the early extracellular electron uptake condition, both planktonic and electrode-attached cells were collected and preserved for RNA sequencing. Bioelectrochemical cells were set up as described below. To obtain enough biomass for RNA isolation, each replicate was comprised of 6 individual bioelectrochemical cells pooled together; that is, 18 reactors were required to obtain enough biomass for triplicates of this condition. Reactors were individually disconnected from the potentiostat to minimize the possibility of gene expression changes under open circuit conditions during sample processing. Then, 500 μL of the planktonic fraction from each reactor was collected, pooled (six reactors per replicate), and processed with the RNAProtect Bacteria reagent as above while maintaining 1:2 sample:RNAProtect Bacteria reagent volume ratios. To collect cathode-attached biofilms, the remaining planktonic fraction in bioelectrochemical cells was removed by decanting and/or pipetting. Then, 2 mL of RNAProtect Bacteria reagent was immediately added to the electrode surface, and the surface-attached biofilm was scraped using a sterile cell scraper. Biofilm samples were pooled (six reactors per replicate, same provenance as planktonic fractions) and processed as above. Cell pellets were immediately frozen at −80°C until RNA purification

Finally, for the sustained extracellular electron uptake condition, triplicate samples, each comprised of six individual replicate bioelectrochemical cells, were processed as the early EEU condition, and cell pellets were immediately frozen following processing at −80°C until RNA purification.

### RNA purification, sequencing, and data analysis

RNA was isolated from frozen, RNAProtect-treated cell pellets following the Enzymatic Lysis and Proteinase K Digestion of Bacteria protocol (Protocol 4) located in the RNAProtect Bacteria Reagent (Qiagen) handbook. RNA was then purified with the RNeasy Mini Kit (Qiagen), with the addition of ß-mercaptoethanol to Buffer RLT immediately before use and inclusion of the optional on-column DNase treatment. RNA concentrations were measured for all extractions using a Qubit fluorometer and Qubit RNA HS Assay Kit (Thermo Fisher Scientific). RNA extracts were frozen at −80°C.

Frozen RNA extracts were shipped on dry ice to SeqCenter (Pittsburgh, PA) for cDNA synthesis, library preparation, and RNA sequencing. At SeqCenter, samples were treated with RNase-free DNase (Invitrogen), RNA was converted to cDNA, and sequencing libraries were prepared with Illumina’s Stranded Total RNA Prep Ligation with Ribo-Zero Plus kit with 10 bp unique dual indices. Sequencing was carried out on a NovaSeq X Plus sequencer, which produced 2 × 151 bp reads. Demultiplexing, quality control, and adapter trimming were performed with bcl-convert v4.1.5.

Sequencing read quality was assessed with FastQC 0.12.1 (28). Reads were then quality filtered using Trimmomatic 0.39 (29), with the following options: LEADING:20 TRAILING:20 HEADCROP:10 SLIDINGWINDOW:4:20 MINLEN:20. Trimmed reads were mapped to the *Thioclava electrotropha* ElOx9^T^ reference genome (NCBI Accession no. GCF_002085925.2) with bowtie2 2.5.1 (30) in sensitive mode. SAMtools 1.19.2 was used for sam/bam file conversions, sorting, and indexing (31). Mapped reads were then quantified to estimate RNA abundance using the featureCounts command in the Subread package version 2.0.6 (32) with arguments -p and -s 2. Transcriptomic sequencing statistics and read mapping rates are presented in Table S1. Resultant count matrices were then imported into R Studio for normalization and differential expression analysis (33) with DESeq2 version 1.42.1 (34). In pairwise comparisons between experimental conditions, genes with adjusted *P*-values (Benjamini-Hochberg method) ≤ 0.01 and |log_2_FC| ≥ 1 were considered differentially expressed. Transcriptomic data were visualized in R using packages ggplot2 version 3.5.1 (35) and ComplexHeatmap version 2.18.0 (36). For additional context, the genome was queried for putative *c-*type cytochromes (those genes containing putative *c*-type heme-binding motifs (CX_2-4_CH)) and were identified using Prosite (37). PSORTb 3.0.3 was used to predict the subcellular localization of all protein-coding genes in the genome (38).

### Reactor setup and bioelectrochemical measurements

Bioelectrochemical experiments were performed in standard three-electrode bioelectrochemical cells as previously described (18). The reactor was comprised of a 10.68 cm^2^ indium-tin oxide coated glass electrode (Delta Technologies), a platinum wire counter electrode (Sigma-Aldrich), and an Ag/AgCl (1M KCl) reference electrode (CH Instruments). Reactors were filled with 20 mL of cell resuspensions and bubbled with 0.2 μm-filtered room air to provide oxygen as the terminal electron acceptor. Amperometric and voltammetric measurements were made with either a 16-channel MPG-2 potentiostat (BioLogic) or a 4-channel Squidstat Prime potentiostat (Admiral Instruments).

For transcriptomic experiments, wild-type *Thioclava electrotropha* ElOx9^T^ cells were grown overnight in LBS + Ions broth, pelleted via centrifugation (8,000 × *g*, 5 min), and resuspended in SWB cathode medium (SWB lacking organic carbon, vitamin, and mineral solutions) to a final OD_600_ of 0.2. The cell resuspension was transferred to reactors (20 mL/reactor), and reactors were connected to the potentiostat. For the early extracellular electron uptake condition, the working electrode was poised at an electron-donating potential of −278 mV vs SHE for 24 h. A cathodic potential of −278 mV vs SHE was chosen as this is just below the calculated redox potential for S^0^ oxidation in seawater at pH 8 but well above the potential necessary for H_2_ evolution to prevent H_2_ from serving as an electron donor to the cells (18, 19). For the sustained extracellular electron uptake condition, the working electrode was poised at −278 mV vs SHE for ~6 days, and current was measured (chronoamperometry [CA]). After 60 h of chronoamperometry, cyclic voltammetry (CV) was performed to assess reactor health. The working potential was cycled between −378 and 422 mV vs SHE three times at a scan rate of 1 mV/s for the Biologic potentiostat or 2.4 mV/s for the Squidstat Prime potentiostat.

For experiments determining the capacity for various *Thioclava electrotropha* strains to oxidize a cathode poised at an electron-donating potential, strains were grown overnight in SWB + Acetate medium. Kanamycin (50 mg/L) was added to the medium for plasmid maintenance in strains harboring pBBR1MCS-2 vectors. Cell-free bioelectrochemical cells containing sterile SWB cathode medium, and kanamycin as required, were set up as described above. CA was performed as above until current measurements stabilized, typically 30 min. Linear sweep voltammetry (LSV) was then performed by scanning the working potential from +422 mV to −578 mV vs SHE at a scan rate of 1 mV/s. Three sweeps were performed. The abiotic LSVs were used for background subtraction. After abiotic LSVs were complete and current stabilized, cells from overnight cultures were pelleted via centrifugation (8,000 × *g*, 5 min) and resuspended in the bioelectrochemical cells to a final OD_600_ of 0.2. CA was performed for approximately 6 days followed by LSV.

To determine the amount of current consumption that was due to biological processes, NaCN was added to a final concentration of 10 mM at the end of CA experiments. We define biological current as the difference in average current consumption in the 30 min preceding NaCN addition and average current consumption in the 30 min following NaCN addition. A minimum of four replicates were performed for each strain. Student’s *t*-tests were used to determine if the average biological currents of deletion and complementation strains differed from wild type.

### Construction of gene deletion and complementation mutants

*T. electrotropha* in-frame gene deletion strains ARR288, ARR322, and ARR324 were constructed following Saltikov and Newman (26) with modifications to improve merodiploid resolution rates. Briefly, 1-kb regions flanking the target gene(s) were PCR amplified using the primers listed in Table S2 using NEB Q5 high-fidelity DNA polymerase. PCR amplicons were size selected via agarose gel electrophoresis (0.8% w/v agarose) and gel extracted using the NEB Monarch gel extraction kit. The pSMV3 vector was isolated from overnight cultures of *E. coli* WM3064 using the NEB Monarch plasmid miniprep kit, digested with *Sac*I-HF and *Bam*HI-HF (NEB), size selected, and gel extracted. DNA concentrations of PCR fragments and digested, cleaned pSMV3 vector backbone were quantified with a Qubit fluorometer and 1× dsDNA HS assay kit (Invitrogen) and assembled with NEB HiFi DNA Assembly kit per the manufacturer’s guidelines. DNA assembly reactions were then processed through the NEB Monarch gel extraction kit directly to remove reaction components and other inhibitors that inhibit transformation. Approximately 10 ng of the cleaned assembly was transformed into *E. coli* WM3064 via electroporation (1-mm cuvette gap, 2.1 kV). Transformants were verified for correct assembly by colony PCR with M13 primers by determining insert length. Confirmed plasmids were transferred to *T. electrotropha* by conjugation (1:1 donor to recipient ratio, 16 h mating on an LBS + Ions + DAP plate, 30°C). Transconjugants containing the integrated mutagenesis vector were selected for on LBS + Ions agar plates supplemented with 50 mg/L kanamycin. Transconjugants were then streaked onto SWB plates containing decreased NaCl (0.5% NaCl versus 2% in standard SWB) and grown heterotrophically with 10% w/v sucrose as the sole carbon source at 22°C for 7–10 days, or until colonies arose. Colonies were then replica plated onto LBS + Ions agar and LBS + Ions + kanamycin agar. Kanamycin-sensitive colonies were then screened by PCR for their respective gene deletions. Gene deletion mutants were then verified via whole-genome sequencing as described below.

Complementation plasmids for each gene deletion were constructed by cloning the ORF(s) in the direction of the *lac* promoter in the broad host complementation vector pBBR1MCS-2 (27). pBBR1MCS-2 was isolated from overnight cultures of *E. coli* WM3064, as described above. PCR was used to generate gene fragments containing the deleted gene(s) and flanked with *Bam*HI and *Sac*I sites on the 5′ and 3′ ends, respectively. Isolated vector and PCR products were restriction digested with *Bam*HI-HF and *Sac*I-HF (NEB) and subsequently cleaned and concentrated with the NEB Monarch gel extraction kit. PCR products were then ligated into the digested vector using T4 DNA Ligase (NEB). Ligations were cleaned and concentrated using the NEB Monarch gel extraction kit and transformed into WM3064 via electroporation as described above. Transformants were selected for on LBS + Ions + DAP + 50 mg/L kanamycin plates. Successful ligations were verified by PCR using M13 primers. The verified complementation vectors were conjugated into their respective *T. electrotropha* deletion strain and transconjugants were selected by plating onto LBS + Ions + 50 mg/L Kanamycin agar plates.

### Whole genome sequencing and analysis

Deletion strains were subjected to whole genome sequencing to verify successful gene deletion. Each deletion strain was grown in LBS + Ions broth overnight. DNA was extracted from overnight cultures using the Qiagen DNeasy UltraClean Microbial Kit according to the manufacturer’s instructions. DNA extracts were sent to SeqCenter (Pittsburgh, PA) for library preparation and sequencing. Briefly, sequencing libraries were prepared using the tagmentation-based and PCR-based Illumina DNA Prep kit using custom 10 bp unique dual indexes and selecting a target insert size of 280 bp. No additional DNA fragmentation or size selection steps were performed. The sequencing libraries were sequenced on an Illumina NovaSeq X Plus sequencer, which produced 2 × 151 bp paired-end reads. Demultiplexing and initial quality control and adapter trimming was performed with bcl-convert v4.2.4 (Illumina).

Sequencing read quality was assessed with FastQC 0.12.1 (28). Reads were then quality filtered using Trimmomatic 0.39 (29) with the following options: LEADING:20 TRAILING:20 HEADCROP:10 SLIDINGWINDOW:4:20 MINLEN:100. Trimmed reads were mapped to the *Thioclava electrotropha* ElOx9^T^ reference genome (NCBI Accession no. GCF_002085925.2) with bowtie2 2.5.1 (30) in sensitive mode. Sequencing statistics are presented in Table S3. SAMtools 1.19.2 was used for sam/bam file conversions, sorting, and indexing (31). Sorted and indexed BAM files were visualized against the reference genome using the Integrative Genomics Viewer web application available at igv.org (39). Plots displaying genome annotations and read depth coverage were created using a custom JavaScript-based interactive tool developed with React and HTML5 Canvas (Fig. S1).

### Ion chromatography for anion (sulfate) analysis

Medium samples from the abiotic control, triplicate wild-type sulfur oxidation transcriptome samples, and triplicate ∆*soxCD* (ARR288) sulfur oxidation assays were collected to determine sulfate concentrations via ion chromatography. One milliliter of media from triplicate cultures was collected and passed through 0.2-μm polyethersulfone filters at the time of inoculation (*t* = 0 h) and 4 h post-inoculation (*t* = 4 h). An additional sampling was conducted 24 h post-inoculation for ∆*soxCD* replicates. Samples were diluted 1:5 in MilliQ water and analyzed with a Dionex Aquion Ion Chromatograph (Thermo Fisher Scientific).

### Phylogenetic analysis and codon usage bias analysis

Homologs to PmcA were identified by querying the NCBI blastp nonredundant database (search date: 23 October 2025, Tables S6 and S7). Two phylogenetic trees were constructed: one containing ~40 manually selected representatives from broad phylogenetically distinct lineages and one containing the top ~50 homologs. All sequences used in phylogenetic analyses had >50% amino acid identity and >69% (100 amino acids minimum) query coverage to PmcA. Amino acid sequences were aligned with MUSCLE as implemented in EMBL EBI’s Job Dispatcher (40). RAxML v. 8.2.12 was used to infer phylogenetic relationships using the maximum likelihood method and PROTGAMMAWAG amino acid substitution model. Bootstrap values were calculated based on 100 trees generated. The resulting phylogenetic trees were visualized and annotated with the Interactive Tree of Life (iTOL) program (41).

We evaluated codon usage bias for genes *pmcA* (AKL02_08760), AKL02_08765, AKL02_08770, and AKL02_08775, which corresponds to *T. electrotropha*’s putative cytochrome *c*-multicopper oxidase operon. Relative synonymous codon usage (RSCU) values for each gene and for the genome were calculated using the following formula:


$$RSCU=\;\frac{observed\;frequency\;of\;codon}{expected\;frequency\;of\;codon\;under\;uniform\;usage}$$

The observed frequency of a codon is defined as follows:


$$observed\;frequency=\;\frac{number\;of\;times\;specific\;codon\;appears\;in\;a\;gene}{total\;number\;of\;codons\;encoding\;that\;amino\;acid}$$

The expected frequency of a codon under uniform usage is defined as follows:


$$expected\;frequency=\;\frac{1}{number\;of\;synonymous\;codons\;for\;that\;amino\;acid}$$

For each gene, coding sequences were parsed into non-overlapping triplets starting from the start codon (ATG). Codons were grouped by their corresponding amino acids according to the standard genetic code. For each amino acid with multiple synonymous codons, the observed usage of each codon was compared with the expected usage under random selection.

## RESULTS AND DISCUSSION

### Gene expression profiles differ based on electron source

**Fig 1:**
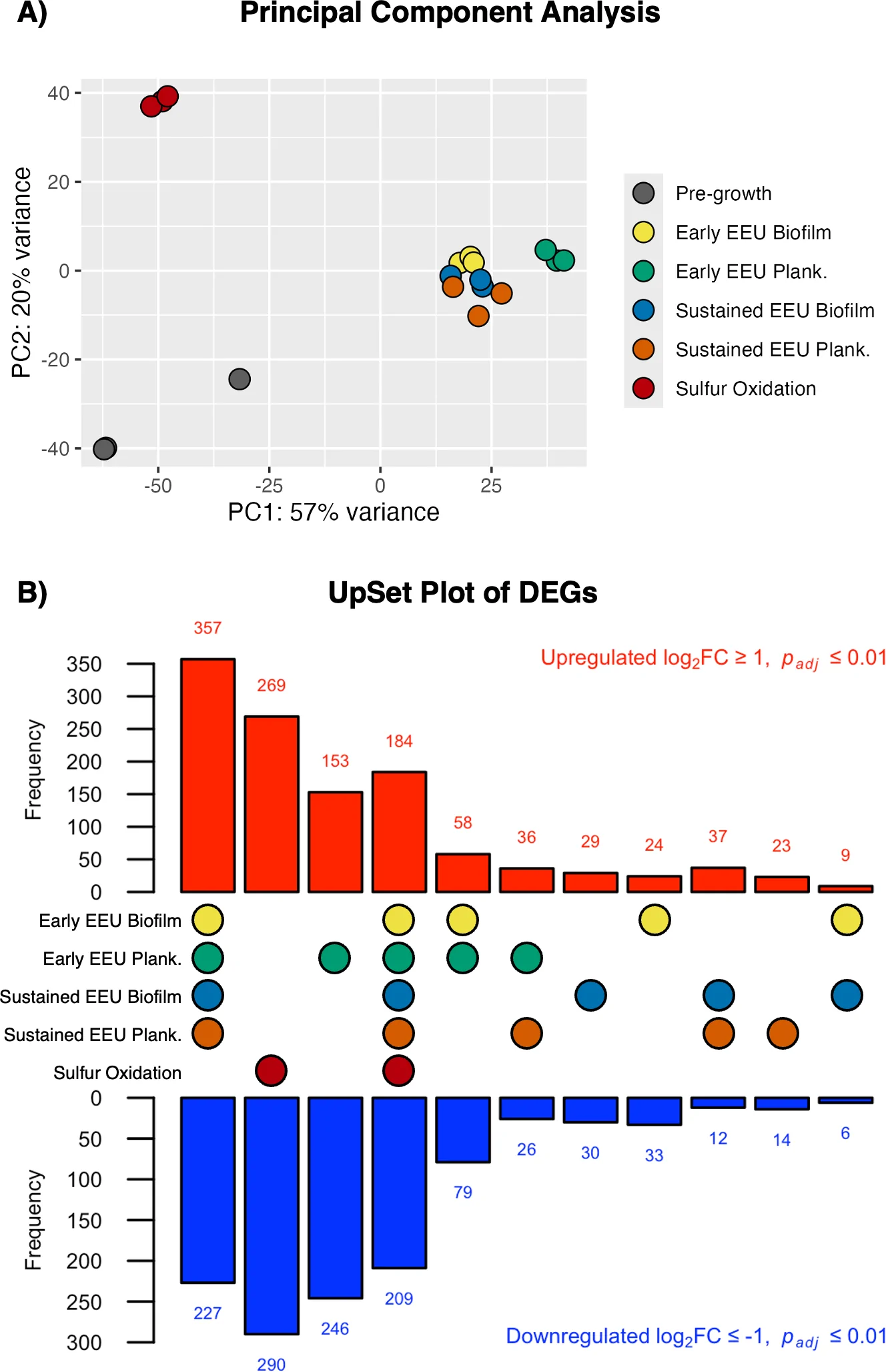
Transcriptome analysis. (**A**) Principal component analysis of RNA-seq data for all biological replicates reveals distinct gene expression profiles for the aerobic heterotrophic pre-growth condition (gray), sulfur oxidation condition (red), and the four EEU sample types (early EEU biofilm – yellow, early EEU planktonic fraction – green, sustained EEU biofilm – blue, sustained EEU planktonic fraction – orange). (**B**) UpSet plot showing the distribution of differentially expressed genes (DEGs) unique to and shared among the sulfur oxidation condition and all EEU conditions collectively, with respect to the pre-growth condition. Upregulated genes are in red, and downregulated genes are in blue (log_2_FoldChange ≥1 or ≤1, respectively, *P*_adj_ ≤ 0.01).

A transcriptomic experiment was conducted to evaluate *T. electrotropha* gene expression under three conditions, each utilizing a different electron source (Fig. 1A). The aerobic heterotrophic pre-growth condition utilized acetate as an organic electron donor, whereas the two chemolithotrophic conditions utilized inorganic electron donors: either reduced sulfur species or an indium-tin oxide electrode poised as a cathode. Transcriptome profiles from each condition formed discrete experimental clusters (Fig. 1A). The two chemolithotrophic conditions were chosen as they utilize two different inorganic electron donors, though they create contrasting chemical microenvironments. Complete oxidation of thiosulfate or elemental sulfur produces sulfuric acid as a byproduct, resulting in acidic conditions (42, 43). In cathode systems, electron uptake likely consumes protons, as one would expect for electrochemical oxygen reduction that generates water, resulting in a localized alkaline microenvironment around the electrode (44, 45). Given the environmental differences between these two conditions, it is not surprising that the overall expression patterns differ distinctly (Fig. 1A).

Differential expression analysis, comparing all transcriptome profiles against the aerobic heterotrophic pre-growth condition, identified more than one thousand significantly differentially expressed genes (DEGs, log_2_FC ≥ |1|, *P*_adj_ ≤ 0.01) in each condition (Table S4; Fig. S2). In line with the clustering patterns observed in the PCA analysis, 584 genes were differentially expressed under all EEU conditions but were not differentially expressed in the sulfur oxidation condition (Fig. 1B). Conversely, 559 DEGs were unique to the sulfur oxidation condition, which highlights the unique transcriptional profiles of these two lithotrophic conditions. These results may suggest that cells employ different mechanisms or strategies for the oxidation of these two inorganic electron donors and/or different strategies for dealing with acid production resulting from sulfur oxidation compared with the alkaline and reducing environment at the cathode. Supporting this is the fact that most of the *sox* sulfur oxidation pathway was upregulated during sulfur oxidation, but only the periplasmic *c-*type cytochrome encoded by *soxX* was upregulated in cathode-oxidizing conditions (all comparisons with respect to pre-growth, Table S4). All other genes in the *sox* pathway were not differentially expressed during cathode oxidation with respect to pre-growth. Previous whole-genome mutational analysis in *Thioclava electrotropha* found that the *sox* sulfur oxidation pathway was not required for cathode oxidation (20), providing further evidence in support of a unique metabolic pathway for cathode oxidation independent of the *sox* system.

All cathode oxidation samples clustered together regardless of experiment duration (early vs sustained) and cell fraction (biofilm vs planktonic cells) (Fig. 1A). The early EEU condition refers to bioelectrochemical cells that were operated for 24 h. The sustained EEU condition refers to bioelectrochemical cells that were operated for approximately 6 days, in line with previous experiments (20). Biofilm samples were taken from biomass attached to the electrodes as opposed to the cells collected from the media suspension. Differences observed in these sample groups were intended to inform gene expression differences that result from transitioning to cathodic conditions, forming a biofilm on the electrode, and/or sustaining these activities.

Overall, we observed modest differences in gene expression profiles across experiment durations and cell fractions, particularly regarding the early EEU planktonic fraction. This sample had the highest number of unique DEGs (399 genes differentially expressed only in this condition, Fig. 1B). Compared with all other cathode samples, the early EEU planktonic fraction had more upregulated genes for transcription (COG category K, 111 genes vs 66–79) and replication, recombination, and repair (L, 90 vs 62–73) (Fig. S3A). The early EEU planktonic fraction exhibited more downregulated genes for translation (J, 119 vs 36–40), energy production (C, 102 vs 68–74), and amino acid transport and metabolism (E, 115 vs 53–76) compared with all other cathode samples (Fig. S3B). Given that planktonic *T. electrotropha* cells are not known to directly oxidize a cathode (21), differences in early EEU biofilm and planktonic gene expression patterns are expected as cells transition to biofilm formation and utilization of a solid-phase electron source. Notably, these differences diminished during sustained EEU, whereby the biofilm and planktonic gene expression profiles were nearly indistinguishable. This potentially indicates that either planktonic cells may be participating in EEU with the cathode-attached biofilms or that biofilms are releasing daughter cells into the planktonic phase. These differences in gene expression patterns during early EEU may inform early adaptations to cathodic conditions; however, given the tight clustering of transcriptome profiles of all cathode samples and low baseMean and/or log_2_FC values for the unique DEGs in the early EEU planktonic sample, these gene expression differences are modest relative to the overall similarity in cathodic transcriptional profiles. Nonetheless, these early EEU samples provide an additional time point in our data set to investigate gene expression dynamics during EEU.

### Putative *c*-type cytochrome gene expression is upregulated during cathode oxidation

**Fig 2:**
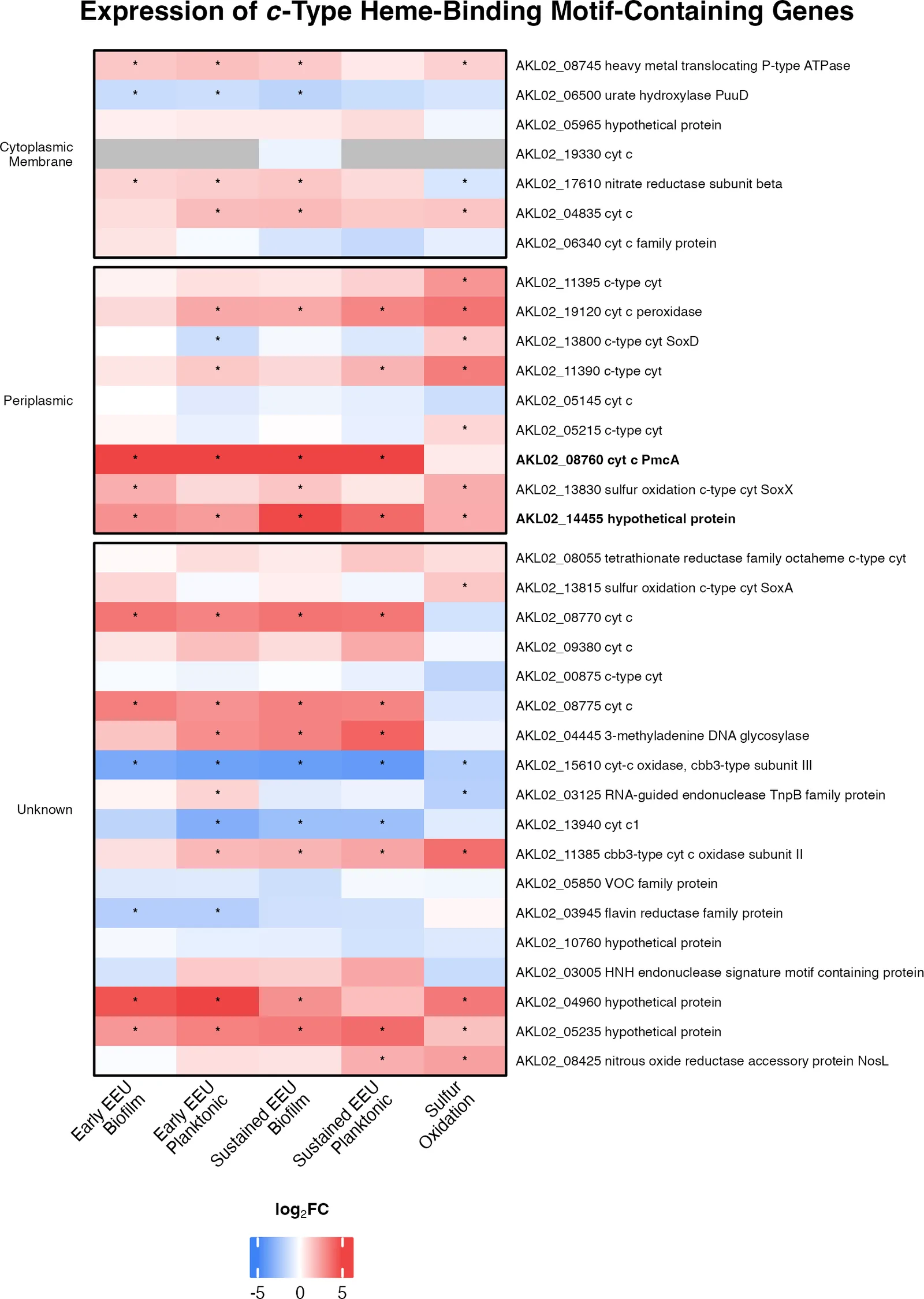
Putative *c-*type cytochromes are upregulated during EEU. Heatmap of DEGs containing putative *c*-type heme-binding motifs predicted to localize to the cellular envelope or with unknown localizations. The log_2_FC values for all genes under all lithotrophic conditions with respect to the pre-growth condition are indicated by cell color, with upregulated genes shown in red and downregulated genes shown in blue. Cells containing an asterisk (*) were statistically significantly differentially regulated (log_2_FoldChange ≥1 or ≤1, *P*_adj_ ≤ 0.01). GenBank locus tag numbers and annotations are listed for all genes. The two genes selected for bioelectrochemical investigation, *pmcA* and hypothetical protein AKL02_14455, are shown in bold. Further details can be found in Table S5.

In bacteria that engage in interfacial extracellular electron transfer, *c*-type cytochromes, particularly those containing multiple heme cofactors, make up the majority of characterized proteins involved in these processes (3, 46, 47). These proteins typically span the inner to outer membrane in Gram-negative bacteria (3, 46). As *Thioclava electrotropha* is a Gram-negative bacterium that has been shown to require direct contact with a cathode for EEU (21), we searched the list of DEGs (relative to aerobic heterotrophic pre-growth) for upregulated genes that are predicted to localize to the cellular envelope and are predicted to contain *c*-type heme binding motifs (Fig. 2; Table S5). To be broadly inclusive of putative cytochromes, genes containing both canonical (CXXCH) and non-canonical (CX_3-4_CH) *c*-type heme binding motifs were included as studies have shown that cytochromes containing atypical *c*-type heme-binding motifs can facilitate EET (48–51).

Gene expression patterns for genes that fit these criteria were remarkably similar between all EEU conditions regardless of time point or cell fraction (Fig. 2). In biofilms from the sustained EEU condition, we identified thirteen genes predicted to contain *c-*type heme-binding motifs that were significantly upregulated and predicted to localize to the cellular envelope, most of which were also upregulated in all other EEU samples. Among all upregulated genes, AKL02_08760 demonstrated the highest fold-change in expression, which increased over 100-fold with respect to pre-growth. Based on RefSeq annotations, this gene encodes a 144 amino acid *c*-type cytochrome that is predicted by pSORTb to localize to the periplasm. Sequence analysis indicates this gene contains a single CXXCH heme-binding motif. Hereafter, we designate AKL02_08760 as *pmcA* for periplasmic monoheme *c*-type cytochrome. A second gene was highly upregulated in all EEU conditions was AKL02_14455, a hypothetical protein containing a single CXXCH heme-binding motif that is also predicted to localize to the periplasm. This gene was upregulated ~8-fold in early EEU biofilms and was upregulated ~30-fold in sustained EEU biofilms with respect to pre-growth. Although the potential role of these genes in EEU is unclear based on sequence analysis alone, the fact that they are two of the most upregulated genes under EEU conditions, they are predicted to localize to the cell envelope, and each contains a putative *c*-type heme-binding motif encouraged us to further investigate the role of these proteins in EEU.

### Development of a genetic system in *Thioclava electrotropha*

To investigate the role of these highly upregulated *c-*type heme-binding motif-containing genes in EEU, we first established a genetic deletion system in *Thioclava electrotropha*. While we have previously demonstrated genetic tractability in this organism (20), clean gene deletion mutants had not yet been constructed. We employed the pSMV3 allelic exchange vector—a suicide plasmid commonly used to generate scarless deletions in other electroactive organisms, such as *Shewanella* species (26). However, we discovered that successful sucrose counterselection was only achievable when *T. electrotropha* was grown heterotrophically in low-salt (0.5% NaCl) medium lacking organic carbon sources other than 10% sucrose, and when grown below the organism’s optimal growth temperature at ≤22°C. Cells consistently evaded counterselection in both complex media (LBS + Ions + 10% sucrose) and defined media (SWB + acetate + 10% sucrose), even when NaCl was adjusted to 0.5% and cells were grown at ≤22°C. These requirements are consistent with established constraints on SacB counterselection: elevated sodium chloride concentrations have been shown to lower SacB activity and/or attenuate sucrose toxicity, and decreased temperatures are thought to improve SacB stability in some bacteria (52). However, the necessity to remove alternative carbon sources is poorly understood. We speculate that access to preferred carbon substrates may decrease sucrose uptake and/or catabolism, potentially limiting the production and accumulation of levan to toxic levels, although this was not directly tested.

We first tested this gene deletion system by targeting part of *T. electrotropha*’s sulfur oxidation system: *soxCD*. SoxCD is a sulfur dehydrogenase that catalyzes the six-electron oxidation from protein-bound sulfane to sulfone (42). Without SoxCD, complete oxidation of S_2_O_3_^2−^ and/or S^0^ would be blocked. This target was selected because the deletion phenotype could be readily screened for in the laboratory, providing a clear and practical phenotypic marker to validate our gene deletion system. Successful deletion of *soxCD* was verified by whole-genome sequencing and alignment to the *Thioclava electrotropha* reference genome (Fig. S1A). Phenotypic screens indicated our scarless ∆*soxCD* deletion mutant was unable to fully oxidize S_2_O_3_^2−^ and/or S^0^ (sulfate concentrations and pH of the medium remained unchanged after 24 h of growth in SWB containing reduced sulfur species), whereas we measured a decrease in pH from 7.2 to ~4 and an increase in sulfate concentrations from 2.0 ± 0.0 mM to 10.6 ± 0.5 mM after just four hours of growth of wild-type cells (data not shown). It is unclear whether the failure of the ∆*soxCD* mutant to produce sulfate reflects a block in S^0^ oxidation, S_2_O_3_^2−^ oxidation, or both. Nevertheless, this proof-of-concept demonstrated that our genetic system could generate scarless deletions in *T. electrotropha*, and we therefore applied this validated approach to construct deletion mutants of the highly upregulated *c-*type heme-binding motif-containing genes previously highlighted (Table 2).

**TABLE 2:** Characteristics of genes targeted for deletion and subsequent bioelectrochemical characterization^*a*^

	*soxCD* (AKL02_13800-13805)	*pmcA* (AKL02_08760)	AKL02_14455
RefSeq annotation	*c-*type cytochrome soxD; sulfite dehydrogenase soxC	Cytochrome *c*	Hypothetical protein
Predicted localization (score)	Periplasmic (10); unknown (4.9)	Periplasmic (9.83)	Periplasmic (9.84)
Heme-binding motifs	CAACH, CKACH; n.d.	CAACH	CHDCH
COG category	C; S	C	G
KEGG Ontology	K22622; K17225	n.a.^*c*^	K03431
Log_2_FC in Biofilm 24h	0.03; 0.27	**7.10^*b*^**	**2.96**
Log_2_FC in Planktonic 24h	**−1.29; −1.10**	**6.45**	**2.66**
Log_2_FC in Biofilm 6d	−0.26; −0.12	**6.68**	**4.92**
Log_2_FC in Planktonic 6d	−0.90; −0.90	**6.62**	**3.98**
Log_2_FC in S Oxidation	**1.40;** 0.98	0.56	**2.20**

^*a*^ Log_2_FC values are with respect to pre-growth.

^*b*^ Bolded log_2_FC values have *P*_adj_ values ≤ 0.01.

^*c*^ n.d., none detected; n.a., none assigned.

### Electrochemical measurements confirm role of PmcA*,* a putative periplasmic *c*-type cytochrome, in EEU

**Fig 3:**
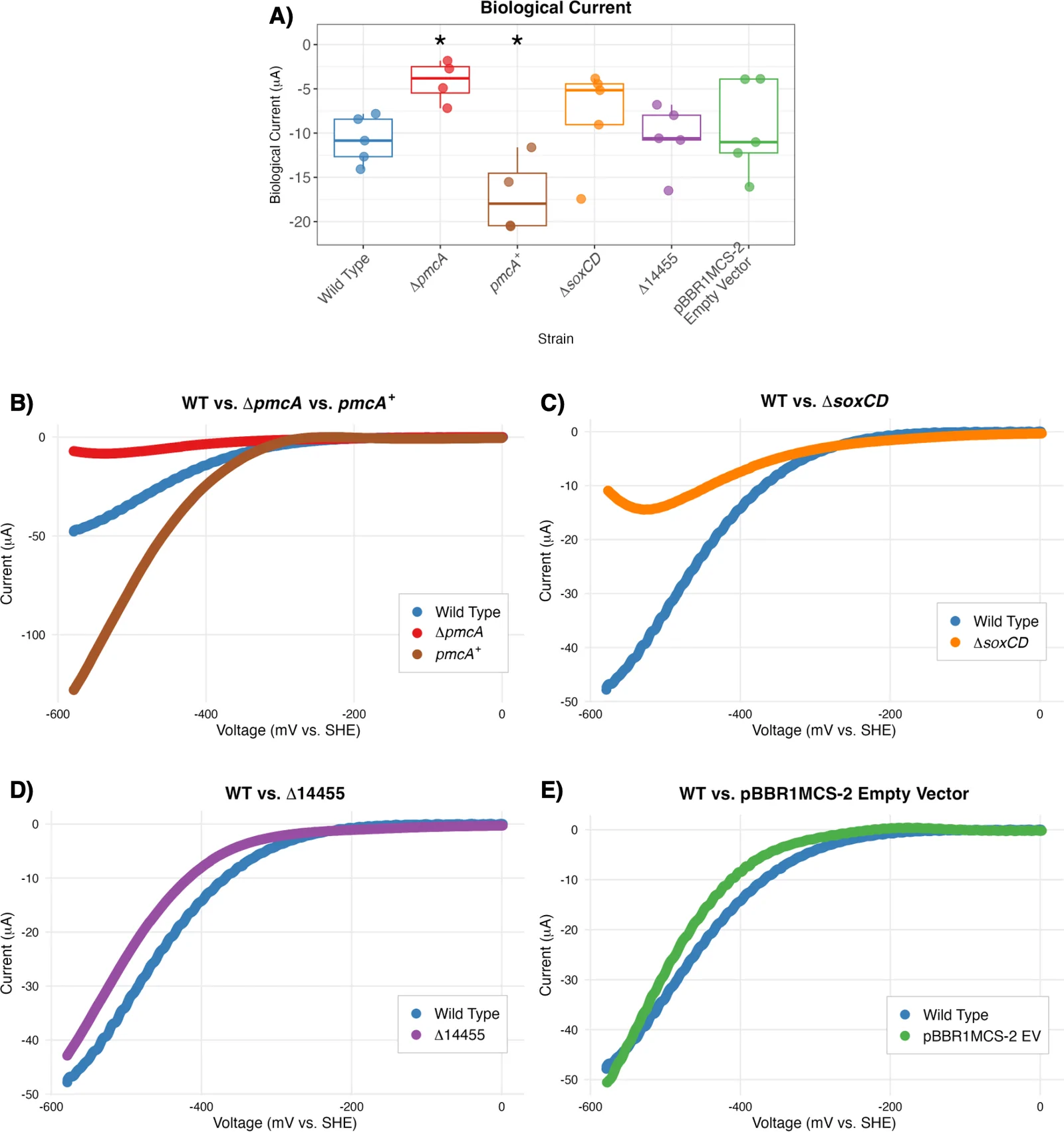
Biological current measurements and catalytic activity for all strains. (**A**) Biological current was determined for all strains (*n* ≥ 4, all strains) after approximately 6 days of continuous chronoamperometry at −278 mV vs SHE (see Fig. S4 for chronoamperometry plots). Biological currents that were significantly different from wild type (*P* < 0.05) are indicated with an asterisk (*). (**B–E**) Representative background-subtracted linear sweep voltammograms (LSVs) of all deletion and complementation strains compared with wild type. (**B**) WT vs ∆*pmcA* vs *pmcA^+^* LSVs show a decrease in catalysis in the deletion mutant that is rescued upon complementation, outperforming the catalytic activity of wild type. (**C**) WT vs ∆*soxCD* LSV shows decreased catalysis in the deletion mutant. (**D**) WT vs ∆14455 LSVs show no difference in catalysis between the two strains. (**E**) WT vs pBBR1MCS-2 empty vector LSV shows no difference in catalysis between the two strains.

We constructed scarless in-frame gene deletion mutants for *pmcA* (Fig. S1B) and AKL02_14455 (Fig. S1C) and tested these mutants electrochemically compared with wild type (Fig. 3). To quantify the amount of current consumption that was due to biological processes in both the mutants and wild-type strains, cyanide was added to reactors to measure abiotic current. We refer to the difference in current consumption pre- and post-cyanide addition as biological current (Fig. S4). Each mutant was tested in at least four replicate experiments.

In the *pmcA* deletion strain, a statistically significant decrease in biological current consumption was measured compared with wild type (Fig. 3A). Linear sweep voltammetry (LSV) was then performed to investigate the electrochemical behavior of each strain, by showing the onset potential of electron uptake for each mutant and the degree of change in electron uptake capacity with increasing voltage (metrics of catalytic activity). Background-subtracted LSV measurements showed that capacity for catalysis was nearly abolished in the ∆*pmcA* strain (Fig. 3B). Interestingly, upon complementation, we measured a statistically significant increase in current consumption (Fig. 3A) and increased catalysis in LSV analysis (Fig. 3B). The enhanced EEU capacity observed upon complementation, exceeding wild-type levels, suggests that altering *pmcA* expression results in enhanced extracellular electron transfer. These findings warrant further investigation into if and how protein abundance is altered under these conditions, and whether modulating expression of *pmcA* affects EEU rates in *T. electrotropha*.

We also measured a modest decrease in electrode oxidation along with decreased catalysis via LSV in the ∆*soxCD* mutant (Fig. 3A and C). While this decrease in biologic current consumption was not statistically significant, it contrasts with observations in *S. oneidensis,* where deletion of periplasmic cytochromes not directly involved in EET led to increased EET rates due to reduced competition for electrons (53). Whether this reflects a minor contribution of SoxCD to EEU, decreased fitness of the mutant, or a change in the architecture of the periplasm that impacts EEU negatively remains unclear. Given that *soxCD* was not differentially expressed with respect to pre-growth and the EEU phenotype of the ∆*soxCD* mutant, ∆*soxCD* was not investigated further. Similarly, we measured no decrease in electrode oxidation capacity or catalysis for the AKL02_14455 deletion strain (Fig. 3A and D). Although this gene was upregulated during EEU, bioelectrochemical measurements indicated that this gene is unlikely to be directly involved in extracellular electron uptake under the experimental conditions tested.

### PmcA homologs are broadly distributed throughout the *Alphaproteobacteria* and in other bacterial phyla

**Fig 4:**
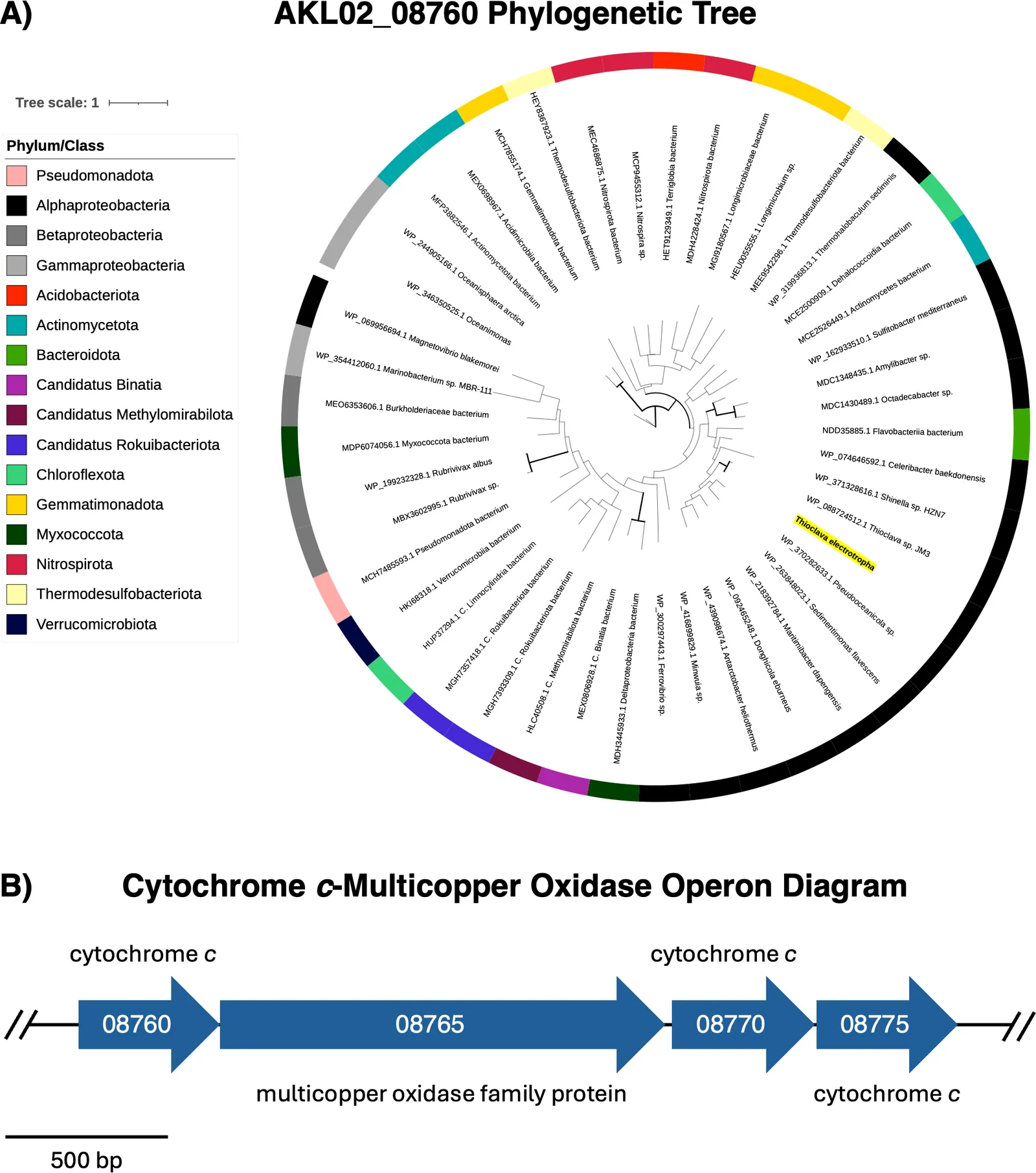
PmcA phylogeny and organization of the putative cytochrome *c*-multicopper oxidase operon. (**A**) The phylogenetic tree was constructed from a manually curated representative selection of homologs to PmcA in the NCBI blastp nonredundant database (queried 23 October 2025, sequences and metadata available in Table S7). All sequences selected for inclusion had ≥50% amino acid identity and ≥69% query coverage (100 amino acids) compared with PmcA. Amino acid sequence alignments were generated using MUSCLE. A best scoring maximum likelihood tree was generated with RAxML v 8.2.12 from 100 bootstrap replicates to identify the optimal tree. The tree was annotated using the interactive tree of life tool (https://itol.embl.de). Branch thickness corresponds to bootstrap support, with thick branches exhibiting 75%–100% bootstrap support. Leaf labels include accession number and lowest taxonomic assignment. Colors in the ring surrounding the tree indicate class-level taxonomic assignments for representatives within the Pseudomonadota when possible, and phylum-level phylogeny for all others. *Thioclava electrotropha* is indicated by yellow highlighting. (**B**) Gene diagram showing the organization of the putative cytochrome *c*-multicopper oxidase operon in *Thioclava electrotropha*. The operon encodes a putative periplasmic monoheme c-type cytochrome PmcA (AKL02_08760), a multicopper oxidase family protein (AKL02_08765), and two additional putative c-type cytochromes (AKL02_08770 and AKL02_08775). Scale bar represents 500 bp.

Phylogenetic analysis of PmcA revealed remarkably broad conservation across diverse bacterial lineages (Fig. 4A; Tables S6 and S7). Homologs are widely distributed among the *Alphaproteobacteria*, other classes of *Pseudomonadota*, and multiple other phyla. In fact, a protein BLAST search of NCBI’s nonredundant protein database identified 2,587 homologs with >50% amino acid sequence identity and >69% coverage (100 amino acids minimum) to PmcA (Table S6). A subset of these sequences was selected to construct a phylogenetic tree showcasing the phylogenetic diversity of organisms harboring homologs to PmcA (Fig. 4; Table S7). Importantly, the region surrounding the heme-binding motif was conserved across all sequences used in phylogenetic reconstruction, suggesting functional constraint on this critical domain.

The phylogenetic distribution of PmcA homologs is inconsistent with vertical inheritance and instead supports horizontal gene transfer. Notably, the gene shows a patchy distribution within major taxonomic groups. For example, homologs are present among some *Alphaproteobacteria*, but their distribution is inconsistent with their phylogeny. PmcA homologs in other EET-capable *Thioclava* species (*T. atlantica* and *T. dalianensis*) (19) showed lower amino acid sequence similarity to PmcA in *T. electrotropha* than several more phylogenetically distant genera (Table S6). Further, homologs appear sporadically across phylogenetically distant lineages of both Gram-positive and Gram-negative bacteria. This distribution pattern, where closely related species differ in gene presence while distantly related organisms share the gene, is characteristic of acquisition by horizontal gene transfer rather than vertical inheritance from a common ancestor. The maintenance of this gene across such diverse recipients suggests that it may confer a selective advantage in specific environmental niches or metabolic contexts.

Horizontal gene transfer of electron transport components, including those involved in EET, has been documented in other electroactive bacteria. For example, comparative genomic analysis revealed that key components of the *mtr* pathway in *Shewanella* show evidence of both vertical and horizontal transmission across diverse bacterial lineages (54). Similarly, the extracellular electron transfer machinery in *Geobacter* species shows phylogenetic patterns consistent with horizontal acquisition of cytochrome genes (23). These observations may provide additional evidence that lateral gene transfer is an important mechanism shaping the distribution of EET capabilities across bacterial lineages. The broad conservation of PmcA across 2,587 bacterial genomes provides a foundation for investigating EEU capacity in other lineages and could reveal whether this cytochrome represents a widespread mechanism for extracellular electron uptake.

Horizontally transferred genes often exhibit suboptimal expression in their new host due to incompatibility between the foreign gene’s regulatory sequences and the recipient’s transcriptional machinery, as well as codon usage bias that mismatches with the host’s tRNA pool (55). Over time, the process of amelioration, the gradual evolutionary process by which horizontally acquired genes adapt to their new genomic context, would be expected to optimize expression of proteins important for function (56). The atypical codon usage patterns observed in *pmcA* (discussed in detail below) suggest that this gene has potentially been recently acquired and has not fully ameliorated to *T. electrotropha*’s biosynthetic context, which may result in decreased transcriptional/translational efficiency and lower steady-state protein levels. Increased electron transfer rates measured in the *pmcA*^+^ complementation strain provide further evidence of suboptimal native expression, which may be due to incomplete amelioration of this potentially recently acquired gene. However, we cannot rule out other explanations, and further work is needed to characterize the relationship between *pmcA* expression and EEU activity.

### Evidence for horizontal transfer of the putative cytochrome *c*-multicopper oxidase operon

MetaCyc analysis (57) predicted that *pmcA* belongs to a four-gene operon (AKL02_08760 through AKL02_08775) that encodes a cytochrome *c*-multicopper oxidase system (Fig. 4B). In addition to PmcA, this operon is also predicted to encode a multicopper oxidase family protein and two additional putative *c*-type cytochromes. Transcriptional analysis supported this operon prediction, as expression of all four genes was significantly upregulated and showed coordinated upregulation across experimental conditions specifically during EEU relative to pre-growth (Fig. 2; Table S4). However, since the pSORTb predicted localizations for putative *c*-type cytochromes AKL02_08770 and AKL02_08775 were unknown, these targets were excluded from further genetic and bioelectrochemical analysis in this study. Also noteworthy is a MerR transcriptional regulator located upstream of the cytochrome *c*-multicopper oxidase operon (AKL02_08750), which was strongly upregulated during EEU (log_2_FC = 4.3–5.5 with respect to pre-growth) but also during sulfur oxidation (log_2_FC = 2.3). MerR family regulators typically respond to metal ions or redox signals (58, 59), suggesting that expression of the operon may be controlled by a metal- or redox-sensitive regulatory circuit. However, as the operon was specifically upregulated only during EEU, MerR upregulation alone is insufficient to explain operon induction, suggesting that EEU-specific regulatory inputs are required, the nature of which remains an important open question.

Within this operon, the multicopper oxidase gene belongs to a protein family that has been implicated in reductive and oxidative EET in other prokaryotes. Multicopper oxidases are well-documented catalysts of Mn^2+^ and Mn^3+^ oxidation in a variety of bacteria (60–62). In *Geobacter sulfurreducens,* multicopper oxidases may be important for reductive EET to iron (III) oxides (63, 64). In *Sideroxydans lithotrophicus* ES-1, three copper-containing proteins, including two encoding multicopper oxidase motifs, were highly expressed during magnetite oxidation (13). In the archaean *Metallosphaera yellowstonensis*, a putative multicopper oxidase gene was highly expressed during growth on Fe(II)-ferrihydrite and pyrite, suggesting a possible role of this gene in Fe(II) oxidation (65). More recently, a recombinant FtsP/CopA-like multicopper oxidase from *Georhizobium sp*. MAB10 was shown to oxidize dissolved Mn^2+^ *in vitro* and was predicted to be involved in oxidative EET *in vivo,* although this has yet to be confirmed genetically (66). However, whether this putative multicopper oxidase contributes to cathode oxidation in *T. electrotropha* remains to be determined. The two monoheme *c*-type cytochromes downstream of the multicopper oxidase lack characterized homologs and do not belong to well-studied cytochrome families previously implicated in extracellular electron transfer (20), highlighting the need for further investigation into the role of this entire operon in EEU.

As with *pmcA* discussed in the previous section, several lines of evidence suggest that this entire operon may have been acquired through horizontal gene transfer. In *T. electrotropha*, this operon exhibited a higher GC content compared with the genome average (66.22% vs 63.87%, respectively), an increase that falls within the range typically associated with HGT events where horizontally transferred genes often display atypical GC content relative to the recipient genome (67, 68). However, GC content alone is not a strong indicator of HGT events, as intrinsic genome variability (e.g., non-uniform GC content) can often explain deviations in GC content of individual genes, operons, or regions of the genome. Therefore, additional analysis evaluating codon usage was performed.

Relative synonymous codon usage (RSCU) analysis provided additional evidence for the potential foreign origin of this operon. While codon usage-based methods for horizontal gene transfer (HGT) detection are known to generate both false positives and negatives (69), our analysis revealed several signatures consistent with foreign gene origin. RSCU analysis quantifies deviations from expected codon frequencies relative to random (equal) usage, where values >1.0 indicate overrepresentation and values <1.0 indicate underrepresentation of specific codons (70). Large deviations in RSCU values between individual genes and the whole genome can indicate HGT events or selection pressure. RSCU analysis (Table S8) revealed significant deviations from genome-wide codon preferences across all operon genes. *pmcA* showed the strongest evidence for foreign origin, with large RSCU differences for 14 codons and an average absolute RSCU difference of 0.563 among all codons. The two other cytochrome *c* genes in the operon (AKL02_08770 and AKL02_08775) exhibited high overall RSCU deviations (0.499 and 0.483, respectively), indicative of potential foreign origin or HGT. However, the multicopper oxidase gene (AKL02_08775) had a low overall absolute RSCU difference (0.289) compared with the cytochrome *c* genes, and no codons were designated as “Strongly Over/Underused,” which may suggest that this gene is subject to stronger selection or functional constraints. However, the average absolute RSCU deviation of the operon was 0.459, which provides strong evidence for HGT of this operon.

The coordinated regulation, atypical codon usage patterns, and functional coherence of this four-gene operon, along with the phylogenetic incongruence of PmcA, provide evidence to suggest that the entire cytochrome *c*-multicopper oxidase system in *Thioclava electrotropha* was acquired through horizontal gene transfer. While we propose that this putative periplasmic *c*-type cytochrome plays a role in EEU in *T. electrotropha,* whether acquisition of this operon alone confers capacity for EEU is unclear. Although 2,587 instances of homologs to PmcA have been found across broad phylogenetic groups, the capacity for extracellular electron uptake has only been confirmed in two additional species beyond *T. electrotropha*: *T. atlantica* and *T. dalianensis*. Whether their PmcA homologs also confer EEU capacity remains to be determined. It is plausible that PmcA participates in other periplasmic electron transfer processes beyond EEU, as has been shown for small tetraheme cytochromes (STCs) and FccA in *Shewanella oneidensis* MR-1, where both proteins serve as shared electron carriers across multiple anaerobic respiratory pathways, including EET (71, 72). We note that *Thioclava indica* is capable of EEU despite lacking this cytochrome *c*-multicopper oxidase operon, suggesting that EEU capacity may include other components more broadly conserved in the *Thioclava*. Further work is needed to evaluate whether homologs of PmcA are associated with EEU capacity in other organisms, and to also investigate the role of all genes in the cytochrome *c*-multicopper oxidase operon in EEU in *T. electrotropha*.

### Conclusion

Extracellular electron uptake enables some microorganisms to access energy from solid-phase electron donors, yet the molecular machinery underlying this process remains poorly understood outside of a few model systems. This work establishes PmcA, a putative periplasmic monoheme *c*-type cytochrome, as an important component of EEU in *Thioclava electrotropha* — an organism that lacks homologs to characterized EET proteins. The enhanced EEU capacity measured upon complementation, exceeding wild-type levels, suggests that native expression of this cytochrome may limit EEU rates, though further investigation is needed to fully characterize this relationship. However, the retention of residual EEU activity in the *pmcA* deletion mutant suggests that PmcA is important but not solely responsible for EEU in *T. electrotropha*. Additional potentially redundant mechanisms, such as other uncharacterized periplasmic *c*-type cytochromes or pathways that are independent of *c*-type cytochromes entirely, may facilitate EEU in this organism. As industrial applications of microbial electrosynthesis and bioremediation expand, understanding and optimizing the factors that control EEU rates will be essential for improving efficiency and expanding the diversity of organisms suitable for these technologies.

The broad phylogenetic distribution of PmcA homologs across more than 2,500 species spanning multiple bacterial phyla raises the possibility that EEU capacity may be more widespread than currently appreciated. Combined with evidence supporting acquisition of the cytochrome *c-*multicopper oxidase operon through horizontal gene transfer, these findings suggest that lateral genetic exchange may serve as an important mechanism for disseminating EEU capacity across diverse bacterial lineages — paralleling observations for other EET pathways. These findings have potential implications for understanding biogeochemical cycling in electron donor-limited environments, where solid-phase minerals, conductive networks, and even other species of bacteria may serve as critical energy sources or conduits. However, whether the presence of this operon alone confers EEU capacity in other organisms remains to be determined.

Several key questions remain for future investigation. First, biochemical characterization of PmcA is needed to determine its redox properties and electron transfer partners. Second, the remaining genes in the cytochrome *c-*multicopper oxidase operon warrant investigation into their potential roles in EEU. Third, testing whether organisms harboring PmcA homologs have the capacity for EEU would clarify the ecological and biotechnological implications of this gene’s broad distribution. Finally, understanding how this pathway integrates with *T. electrotropha*’s central metabolism may reveal new strategies for optimizing microbial electrosynthesis and other bioelectrochemical applications.

## Data Availability

All raw DNA and RNA sequencing data generated in this study have been deposited in the NCBI Sequence Read Archive and are publicly available under BioProject PRJNA1293320. The *Thioclava electrotropha* ElOx9^T^ reference genome is available from GenBank under accession number GCA_002085925.2.

## SUPPLEMENTAL MATERIAL

Supplemental figures aem.00250-26-s0001.pdf Fig. S1 to S5.

Supplemental tables aem.00250-26-s0002.xlsx Tables S1 to S8.

ASM does not own the copyrights to Supplemental Material that may be linked to, or accessed through, an article. The authors have granted ASM a non-exclusive, world-wide license to publish the Supplemental Material files. Please contact the corresponding author directly for reuse.

## References

[1] Nevin KP, Woodard TL, Franks AE, Summers ZM, Lovley DR. 2010. Microbial electrosynthesis: feeding microbes electricity to convert carbon dioxide and water to multicarbon extracellular organic compounds. mBio 1:e00103-10. doi:10.1128/mBio.00103-10 20714445 PMC2921159

[2] Rabaey K, Rozendal RA. 2010. Microbial electrosynthesis — revisiting the electrical route for microbial production. Nat Rev Microbiol 8:706–716. doi:10.1038/nrmicro2422 20844557

[3] Shi L, Dong H, Reguera G, Beyenal H, Lu A, Liu J, Yu HQ, Fredrickson JK. 2016. Extracellular electron transfer mechanisms between microorganisms and minerals. Nat Rev Microbiol 14:651–662. doi:10.1038/nrmicro.2016.93 27573579

[4] Wang X, Aulenta F, Puig S, Esteve-Núñez A, He Y, Mu Y, Rabaey K. 2020. Microbial electrochemistry for bioremediation. Environ Sci Ecotechnol 1:100013. doi:10.1016/j.ese.2020.100013 36160374 PMC9488016

[5] Kim B, Baek G, Kim C, Lee SY, Yang E, Lee S, Kim T, Nam J-Y, Lee C, Chae K-J, et al.. 2024. Progress and prospects for applications of extracellular electron transport mechanism in environmental biotechnology. ACS EST Eng 4:1520–1539. doi:10.1021/acsestengg.4c00077

[6] Edwards MJ, White GF, Butt JN, Richardson DJ, Clarke TA. 2020. The crystal structure of a biological insulated transmembrane molecular wire. Cell 181:665–673. doi:10.1016/j.cell.2020.03.032 32289252 PMC7198977

[7] Jiao Y, Newman DK. 2007. The *pio* operon is essential for phototrophic Fe(II) oxidation in *Rhodopseudomonas palustris* TIE-1. J Bacteriol 189:1765–1773. doi:10.1128/JB.00776-06 17189359 PMC1855732

[8] Guzman MS, Rengasamy K, Binkley MM, Jones C, Ranaivoarisoa TO, Singh R, Fike DA, Meacham JM, Bose A. 2019. Phototrophic extracellular electron uptake is linked to carbon dioxide fixation in the bacterium *Rhodopseudomonas palustris*. Nat Commun 10:1355. doi:10.1038/s41467-019-09377-6 30902976 PMC6430793

[9] Gupta D, Guzman MS, Bose A. 2020. Extracellular electron uptake by autotrophic microbes: physiological, ecological, and evolutionary implications. J Ind Microbiol Biotechnol 47:863–876. doi:10.1007/s10295-020-02309-0 32930890

[10] Liu J, Wang Z, Belchik SM, Edwards MJ, Liu C, Kennedy DW, Merkley ED, Lipton MS, Butt JN, Richardson DJ, et al.. 2012. Identification and characterization of MtoA: a decaheme c-type cytochrome of the neutrophilic Fe(II)-oxidizing bacterium *Sideroxydans lithotrophicus* ES-1. Front Microbio 3:37. doi:10.3389/fmicb.2012.00037 PMC3274759 22347878

[11] Zhou N, Kupper RJ, Catalano JG, Thompson A, Chan CS. 2022. Biological oxidation of Fe(II)-bearing smectite by microaerophilic iron oxidizer *Sideroxydans lithotrophicus* using dual Mto and Cyc2 iron oxidation pathways. Environ Sci Technol 56:17443–17453. doi:10.1021/acs.est.2c05142 36417801 PMC9731265

[12] Zhou N, Keffer JL, Polson SW, Chan CS. 2022. Unraveling Fe(II)-oxidizing mechanisms in a facultative Fe(II) oxidizer, *Sideroxydans lithotrophicus* strain ES-1, via culturing, transcriptomics, and reverse transcription-quantitative PCR. Appl Environ Microbiol 88:e0159521. doi:10.1128/AEM.01595-21 34788064 PMC8788666

[13] Keffer JL, Zhou N, Rushworth DD, Yu Y, Chan CS. 2025. Microbial magnetite oxidation via MtoAB porin-multiheme cytochrome complex in *Sideroxydans lithotrophicus* ES-1. Appl Environ Microbiol 91:e0186524. doi:10.1128/aem.01865-24 40042276 PMC12016527

[14] Yarzábal A, Brasseur G, Ratouchniak J, Lund K, Lemesle-Meunier D, DeMoss JA, Bonnefoy V. 2002. The high-molecular-weight cytochrome *c* Cyc2 of *Acidithiobacillus ferrooxidans* is an outer membrane protein. J Bacteriol 184:313–317. doi:10.1128/JB.184.1.313-317.2002 11741873 PMC134758

[15] McAllister SM, Polson SW, Butterfield DA, Glazer BT, Sylvan JB, Chan CS. 2020. Validating the Cyc2 neutrophilic iron oxidation pathway using meta-omics of *Zetaproteobacteria* iron mats at marine hydrothermal vents. mSystems 5:e00553-19. doi:10.1128/mSystems.00553-19 PMC7029218 32071158

[16] Keffer JL, McAllister SM, Garber AI, Hallahan BJ, Sutherland MC, Rozovsky S, Chan CS. 2021. Iron oxidation by a fused cytochrome-porin common to diverse iron-oxidizing bacteria. mBio 12:e0107421. doi:10.1128/mBio.01074-21 34311573 PMC8406198

[17] Emerson D, Rentz JA, Lilburn TG, Davis RE, Aldrich H, Chan C, Moyer CL. 2007. A novel lineage of proteobacteria involved in formation of marine Fe-oxidizing microbial mat communities. PLoS One 2:e667. doi:10.1371/journal.pone.0000667 17668050 PMC1930151

[18] Rowe AR, Chellamuthu P, Lam B, Okamoto A, Nealson KH. 2014. Marine sediments microbes capable of electrode oxidation as a surrogate for lithotrophic insoluble substrate metabolism. Front Microbiol 5:784. doi:10.3389/fmicb.2014.00784 25642220 PMC4294203

[19] Chang R, Bird L, Barr C, Osburn M, Wilbanks E, Nealson K, Rowe A. 2018. *Thioclava electrotropha* sp. nov., a versatile electrode and sulfur-oxidizing bacterium from marine sediments. Int J Syst Evol Microbiol 68:1652–1658. doi:10.1099/ijsem.0.002723 29570444

[20] Sackett JD, Kamble N, Leach E, Schuelke T, Wilbanks E, Rowe AR. 2022. Genome-scale mutational analysis of cathode-oxidizing *Thioclava electrotropha* ElOx9^T^. Front Microbiol 13:2067. doi:10.3389/fmicb.2022.909824 PMC9226611 35756027

[21] Karbelkar AA, Rowe AR, El-Naggar MY. 2019. An electrochemical investigation of interfacial electron uptake by the sulfur oxidizing bacterium *Thioclava electrotropha* ElOx9. Electrochim Acta 324:134838. doi:10.1016/j.electacta.2019.134838

[22] Meyer TE, Tsapin AI, Vandenberghe I, de Smet L, Frishman D, Nealson KH, Cusanovich MA, van Beeumen JJ. 2004. Identification of 42 possible cytochrome C genes in the *Shewanella oneidensis* genome and characterization of six soluble cytochromes. OMICS 8:57–77. doi:10.1089/153623104773547499 15107237

[23] Butler JE, Young ND, Lovley DR. 2010. Evolution of electron transfer out of the cell: comparative genomics of six *Geobacter* genomes. BMC Genomics 11:40. doi:10.1186/1471-2164-11-40 20078895 PMC2825233

[24] Moser DP, Nealson KH. 1996. Growth of the facultative anaerobe *Shewanella putrefaciens* by elemental sulfur reduction. Appl Environ Microbiol 62:2100–2105. doi:10.1128/aem.62.6.2100-2105.1996 11536738 PMC167988

[25] Kleinjan WE, de Keizer A, Janssen AJH. 2005. Kinetics of the chemical oxidation of polysulfide anions in aqueous solution. Water Res 39:4093–4100. doi:10.1016/j.watres.2005.08.006 16213542

[26] Saltikov CW, Newman DK. 2003. Genetic identification of a respiratory arsenate reductase. Proc Natl Acad Sci USA 100:10983–10988. doi:10.1073/pnas.1834303100 12939408 PMC196913

[27] Kovach ME, Elzer PH, Steven Hill D, Robertson GT, Farris MA, Roop RM, Peterson KM. 1995. Four new derivatives of the broad-host-range cloning vector pBBR1MCS, carrying different antibiotic-resistance cassettes. Gene 166:175–176. doi:10.1016/0378-1119(95)00584-1 8529885

[28] Andrews S. 2015. FastQC: a quality control tool for high throughput sequence data.

[29] Bolger AM, Lohse M, Usadel B. 2014. Trimmomatic: a flexible trimmer for Illumina sequence data. Bioinformatics 30:2114–2120. doi:10.1093/bioinformatics/btu170 24695404 PMC4103590

[30] Langmead B, Salzberg SL. 2012. Fast gapped-read alignment with Bowtie 2. Nat Methods 9:357–359. doi:10.1038/nmeth.1923 22388286 PMC3322381

[31] Danecek P, Bonfield JK, Liddle J, Marshall J, Ohan V, Pollard MO, Whitwham A, Keane T, McCarthy SA, Davies RM, et al.. 2021. Twelve years of SAMtools and BCFtools. Gigascience 10:giab008. doi:10.1093/gigascience/giab008 33590861 PMC7931819

[32] Liao Y, Smyth GK, Shi W. 2014. featureCounts: an efficient general purpose program for assigning sequence reads to genomic features. Bioinformatics 30:923–930. doi:10.1093/bioinformatics/btt656 24227677

[33] R Core Team. 2021. R: a language and environment for statistical computing. Vienna, Austria. R Foundation for Statistical Computing.

[34] Love MI, Huber W, Anders S. 2014. Moderated estimation of fold change and dispersion for RNA-seq data with DESeq2. Genome Biol 15:550. doi:10.1186/s13059-014-0550-8 25516281 PMC4302049

[35] Wickham H. 2011. ggplot2. WIREs Computational Stats 3:180–185. doi:10.1002/wics.147

[36] Gu Z. 2022. Complex heatmap visualization. iMeta 1:e43. doi:10.1002/imt2.43 38868715 PMC10989952

[37] Hulo N, Bairoch A, Bulliard V, Cerutti L, Castro E, Langendijk-Genevaux PS, Pagni M, Sigrist CJA. 2006. The PROSITE database. Nucleic Acids Res 34:D227–D230. doi:10.1093/nar/gkj063 16381852 PMC1347426

[38] Yu NY, Wagner JR, Laird MR, Melli G, Rey S, Lo R, Dao P, Sahinalp SC, Ester M, Foster LJ, et al.. 2010. PSORTb 3.0: improved protein subcellular localization prediction with refined localization subcategories and predictive capabilities for all prokaryotes. Bioinformatics 26:1608–1615. doi:10.1093/bioinformatics/btq249 20472543 PMC2887053

[39] Robinson JT, Thorvaldsdóttir H, Winckler W, Guttman M, Lander ES, Getz G, Mesirov JP. 2011. Integrative genomics viewer. Nat Biotechnol 29:24–26. doi:10.1038/nbt.1754 21221095 PMC3346182

[40] Madeira F, Madhusoodanan N, Lee J, Eusebi A, Niewielska A, Tivey ARN, Lopez R, Butcher S. 2024. The EMBL-EBI job dispatcher sequence analysis tools framework in 2024. Nucleic Acids Res 52:W521–W525. doi:10.1093/nar/gkae241 38597606 PMC11223882

[41] Letunic I, Bork P. 2007. Interactive tree of life (iTOL): an online tool for phylogenetic tree display and annotation. Bioinformatics 23:127–128. doi:10.1093/bioinformatics/btl529 17050570

[42] Friedrich CG, Rother D, Bardischewsky F, Quentmeier A, Fischer J. 2001. Oxidation of reduced inorganic sulfur compounds by bacteria: emergence of a common mechanism? Appl Environ Microbiol 67:2873–2882. doi:10.1128/AEM.67.7.2873-2882.2001 11425697 PMC92956

[43] Jia T, Niu L, Qi Z, Cong W, Xi J, Yang C, Peng Y. 2025. The biosynthesis, storage and utilization of elemental sulfur: enzymatic pathways, molecular mechanisms, and future perspectives. Crit Rev Environ Sci Technol 55:483–506. doi:10.1080/10643389.2024.2421087

[44] Yuan Y, Zhou S, Tang J. 2013. *In situ* investigation of cathode and local biofilm microenvironments reveals important roles of OH^–^ and oxygen transport in microbial fuel cells. Environ Sci Technol 47:4911–4917. doi:10.1021/es400045s 23537198

[45] Blanchet E, Pécastaings S, Erable B, Roques C, Bergel A. 2014. Protons accumulation during anodic phase turned to advantage for oxygen reduction during cathodic phase in reversible bioelectrodes. Bioresour Technol 173:224–230. doi:10.1016/j.biortech.2014.09.076 25305652

[46] Burton JAJ, Edwards MJ, Richardson DJ, Clarke TA. 2025. Electron transport across bacterial cell envelopes. Annu Rev Biochem 94:89–109. doi:10.1146/annurev-biochem-052621-092202 40096216

[47] Gralnick JA, Bond DR. 2023. Electron transfer beyond the outer membrane: putting electrons to rest. Annu Rev Microbiol 77:517–539. doi:10.1146/annurev-micro-032221-023725 37713456

[48] Nevin KP, Kim B-C, Glaven RH, Johnson JP, Woodard TL, Methé BA, DiDonato RJ, Covalla SF, Franks AE, Liu A, et al.. 2009. Anode biofilm transcriptomics reveals outer surface components essential for high density current production in *Geobacter sulfurreducens* fuel cells. PLoS One 4:e5628. doi:10.1371/journal.pone.0005628 19461962 PMC2680965

[49] Inoue K, Qian X, Morgado L, Kim B-C, Mester T, Izallalen M, Salgueiro CA, Lovley DR. 2010. Purification and characterization of OmcZ, an outer-surface, octaheme *c*-type cytochrome essential for optimal current production by *Geobacter sulfurreducens*. Appl Environ Microbiol 76:3999–4007. doi:10.1128/AEM.00027-10 20400562 PMC2893489

[50] Wang F, Chan CH, Suciu V, Mustafa K, Ammend M, Si D, Hochbaum AI, Egelman EH, Bond DR. 2022. Structure of *Geobacter* OmcZ filaments suggests extracellular cytochrome polymers evolved independently multiple times. eLife 11:e81551. doi:10.7554/eLife.81551 36062910 PMC9473688

[51] Gupta D, Chen K, Elliott SJ, Nayak DD. 2024. MmcA is an electron conduit that facilitates both intracellular and extracellular electron transport in *Methanosarcina acetivorans*. Nat Commun 15:3300. doi:10.1038/s41467-024-47564-2 38632227 PMC11024163

[52] Blomfield IC, Vaughn V, Rest RF, Eisenstein BI. 1991. Allelic exchange in *Escherichia coli* using the *Bacillus subtilis sacB* gene and a temperature-sensitive pSC101 replicon. Mol Microbiol 5:1447–1457. doi:10.1111/j.1365-2958.1991.tb00791.x 1686293

[53] Sun W, Lin Z, Yu Q, Cheng S, Gao H. 2021. Promoting extracellular electron transfer of *Shewanella oneidensis* MR-1 by optimizing the periplasmic cytochrome *c* network. Front Microbiol 12:727709. doi:10.3389/fmicb.2021.727709 34675900 PMC8524038

[54] Baker IR, Conley BE, Gralnick JA, Girguis PR. 2022. Evidence for horizontal and vertical transmission of Mtr-mediated extracellular electron transfer among the *Bacteria*. mBio 13:e02904. doi:10.1128/mbio.02904-21 PMC8805035 35100867

[55] Baltrus DA. 2013. Exploring the costs of horizontal gene transfer. Trends Ecol Evol 28:489–495. doi:10.1016/j.tree.2013.04.002 23706556

[56] Lawrence JG, Ochman H. 1997. Amelioration of bacterial genomes: rates of change and exchange. J Mol Evol 44:383–397. doi:10.1007/pl00006158 9089078

[57] Caspi R, Billington R, Keseler IM, Kothari A, Krummenacker M, Midford PE, Ong WK, Paley S, Subhraveti P, Karp PD. 2020. The MetaCyc database of metabolic pathways and enzymes - a 2019 update. Nucleic Acids Res 48:D445–D453. doi:10.1093/nar/gkz862 31586394 PMC6943030

[58] Brown NL, Stoyanov JV, Kidd SP, Hobman JL. 2003. The MerR family of transcriptional regulators. FEMS Microbiol Rev 27:145–163. doi:10.1016/S0168-6445(03)00051-2 12829265

[59] Tulin G, Figueroa NR, Checa SK, Soncini FC. 2024. The multifarious MerR family of transcriptional regulators. Mol Microbiol 121:230–242. doi:10.1111/mmi.15212 38105009

[60] Dick GJ, Torpey JW, Beveridge TJ, Tebo BM. 2008. Direct identification of a bacterial manganese(II) oxidase, the multicopper oxidase MnxG, from spores of several different marine *Bacillus* species. Appl Environ Microbiol 74:1527–1534. doi:10.1128/AEM.01240-07 18165363 PMC2258647

[61] Butterfield CN, Soldatova AV, Lee S-W, Spiro TG, Tebo BM. 2013. Mn(II,III) oxidation and MnO_2_ mineralization by an expressed bacterial multicopper oxidase. Proc Natl Acad Sci USA 110:11731–11735. doi:10.1073/pnas.1303677110 23818588 PMC3718108

[62] Zhang Z, Zhang Z, Chen H, Liu J, Liu C, Ni H, Zhao C, Ali M, Liu F, Li L. 2015. Surface Mn(II) oxidation actuated by a multicopper oxidase in a soil bacterium leads to the formation of manganese oxide minerals. Sci Rep 5:10895. doi:10.1038/srep10895 26039669 PMC4454072

[63] Holmes DE, Mester T, O’Neil RA, Perpetua LA, Larrahondo MJ, Glaven R, Sharma ML, Ward JE, Nevin KP, Lovley DR. 2008. Genes for two multicopper proteins required for Fe(III) oxide reduction in *Geobacter sulfurreducens* have different expression patterns both in the subsurface and on energy-harvesting electrodes. Microbiology (Reading) 154:1422–1435. doi:10.1099/mic.0.2007/014365-0 18451051

[64] Mehta T, Childers SE, Glaven R, Lovley DR, Mester T. 2006. A putative multicopper protein secreted by an atypical type II secretion system involved in the reduction of insoluble electron acceptors in *Geobacter sulfurreducens*. Microbiology (Reading) 152:2257–2264. doi:10.1099/mic.0.28864-0 16849792

[65] Kozubal MA, Dlakic M, Macur RE, Inskeep WP. 2011. Terminal oxidase diversity and function in “*Metallosphaera yellowstonensis*”: gene expression and protein modeling suggest mechanisms of Fe(II) oxidation in the sulfolobales. Appl Environ Microbiol 77:1844–1853. doi:10.1128/AEM.01646-10 21239558 PMC3067268

[66] Xu X, Zhang L, Song F, Zhang G, Ma L, Yang N. 2025. Genomic insights into the alphaproteobacterium *Georhizobium* sp. MAB10 revealed a pathway of Mn(II) oxidation-coupled anoxygenic photoautotrophy: a novel understanding of the biotic process in deep-sea ferromanganese nodule formation. mBio 16:e0267524. doi:10.1128/mbio.02675-24 39584839 PMC11708043

[67] Zhang R, Ou H-Y, Gao F, Luo H. 2014. Identification of horizontally-transferred genomic islands and genome segmentation points by using the GC profile method. Curr Genomics 15:113–121. doi:10.2174/1389202915999140328163125 24822029 PMC4009839

[68] Ravenhall M, Škunca N, Lassalle F, Dessimoz C. 2015. Inferring horizontal gene transfer. PLoS Comput Biol 11:e1004095. doi:10.1371/journal.pcbi.1004095 26020646 PMC4462595

[69] Friedman R, Ely B. 2012. Codon usage methods for horizontal gene transfer detection generate an abundance of false positive and false negative results. Curr Microbiol 65:639–642. doi:10.1007/s00284-012-0205-5 23010940

[70] Sharp PM, Li W-H. 1987. The codon adaptation index-a measure of directional synonymous codon usage bias, and its potential applications. Nucleic Acids Res 15:1281–1295. doi:10.1093/nar/15.3.1281 3547335 PMC340524

[71] Fonseca BM, Paquete CM, Neto SE, Pacheco I, Soares CM, Louro RO. 2013. Mind the gap: cytochrome interactions reveal electron pathways across the periplasm of *Shewanella oneidensis* MR-1. Biochem J 449:101–108. doi:10.1042/BJ20121467 23067389

[72] Alves MN, Neto SE, Alves AS, Fonseca BM, Carrêlo A, Pacheco I, Paquete CM, Soares CM, Louro RO. 2015. Characterization of the periplasmic redox network that sustains the versatile anaerobic metabolism of *Shewanella oneidensis* MR-1. Front Microbiol 6:665. doi:10.3389/fmicb.2015.00665 26175726 PMC4484225

