# Supplementary material for: Comparative transcriptomics and scarless genome editing uncover a periplasmic c-type cytochrome important for extracellular electron uptake in Thioclava electrotropha ElOx9T
Source: Appl Environ Microbiol. 2026 Jul 28;92(8):e00250-26. doi: 10.1128/aem.00250-26 (PMC13488337; doi:10.1128/aem.00250-26)
Supplement: Supplementary file 1 [file aem.00250-26-s0001.pdf]

Supplemental Information For:

Comparative Transcriptomics and Scarless Genome Editing Uncover a Periplasmic *c*-type Cytochrome Important for Extracellular Electron Uptake in *Thioclava electrotropha* ElOx9^T

Joshua D. Sackett, Leah R. Trutschel, Andrew W. Grenfell, Jeffrey A. Gralnick, and Annette R. Rowe

This file contains Supplemental Figures S1-S5. Supplemental Tables S1-S8 are available in the accompanying spreadsheet file.

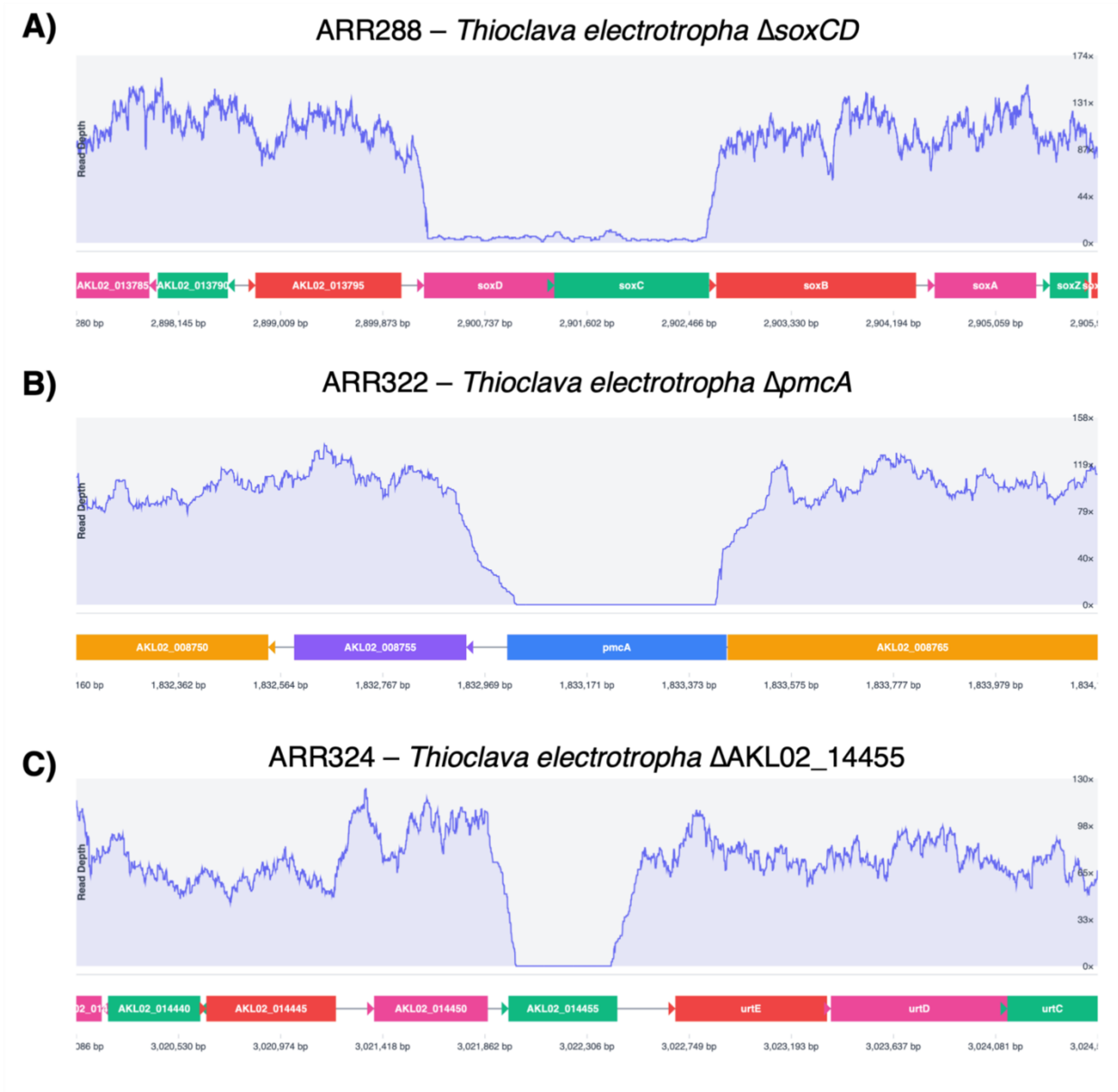

Figure S1. Whole-genome sequencing and read alignments confirm gene deletions. Sequencing reads for each deletion mutant were aligned to the *Thioclava electrotropha* reference genome and read depth histograms were constructed. Read depth is noted on the right of each plot. **A)** Δ*soxCD*. **B)** Δ*pmcA*. **C)** ΔAKL02_14455.

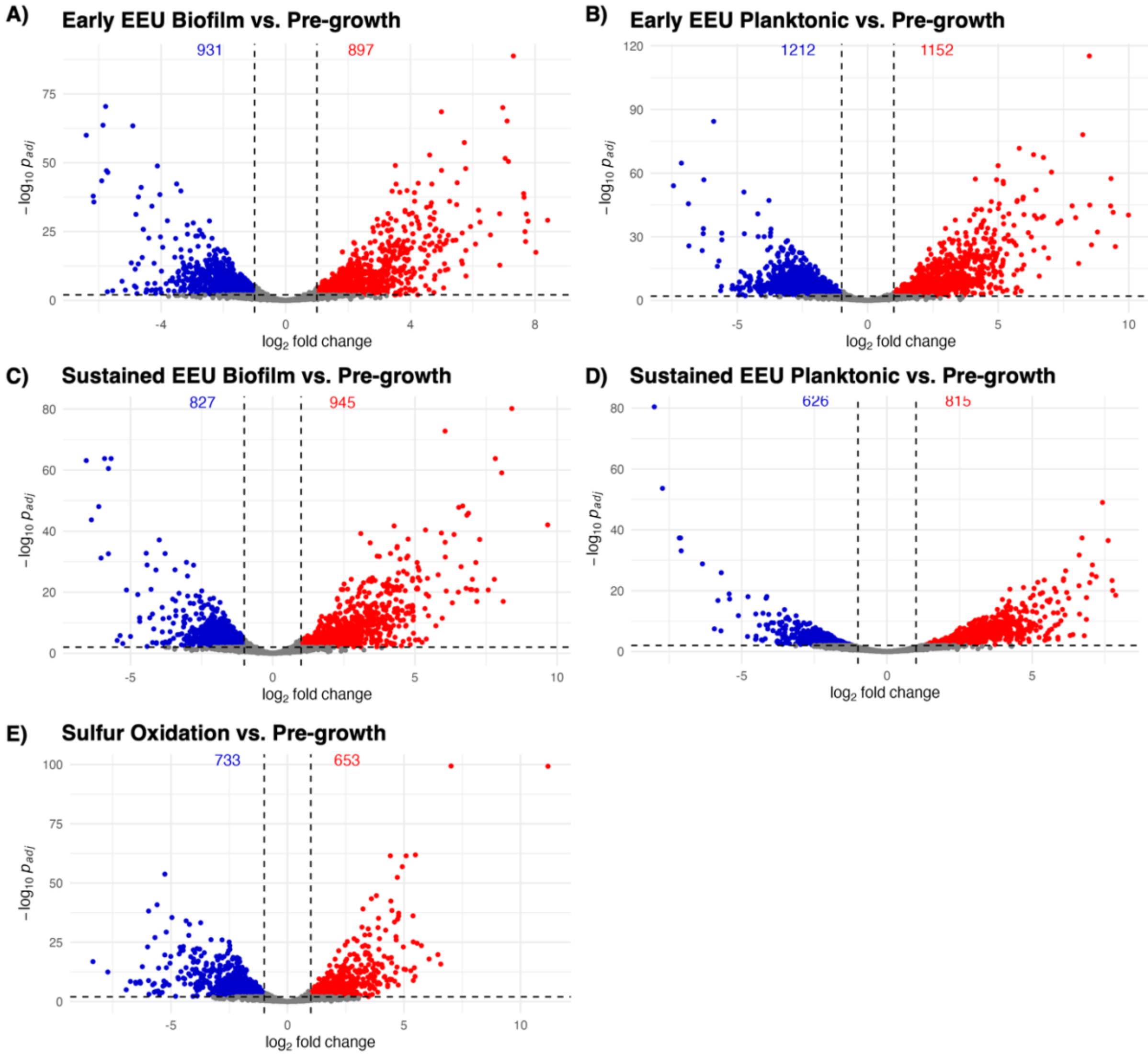

Figure S2. Volcano plots of gene expression profiles with respect to pre-growth. Genes that are upregulated in a condition are in red and genes that are downregulated are in blue (log_2FoldChange ≥ |1|, p_{adj} ≤ 0.01). The number of genes upregulated and downregulated for a particular condition are noted in red and blue text, respectively. **A)** Early EEU biofilm. **B)** Early EEU planktonic fraction. **C)** Sustained EEU biofilm. **D)** Sustained EEU planktonic fraction. **E)** Sulfur oxidation condition.

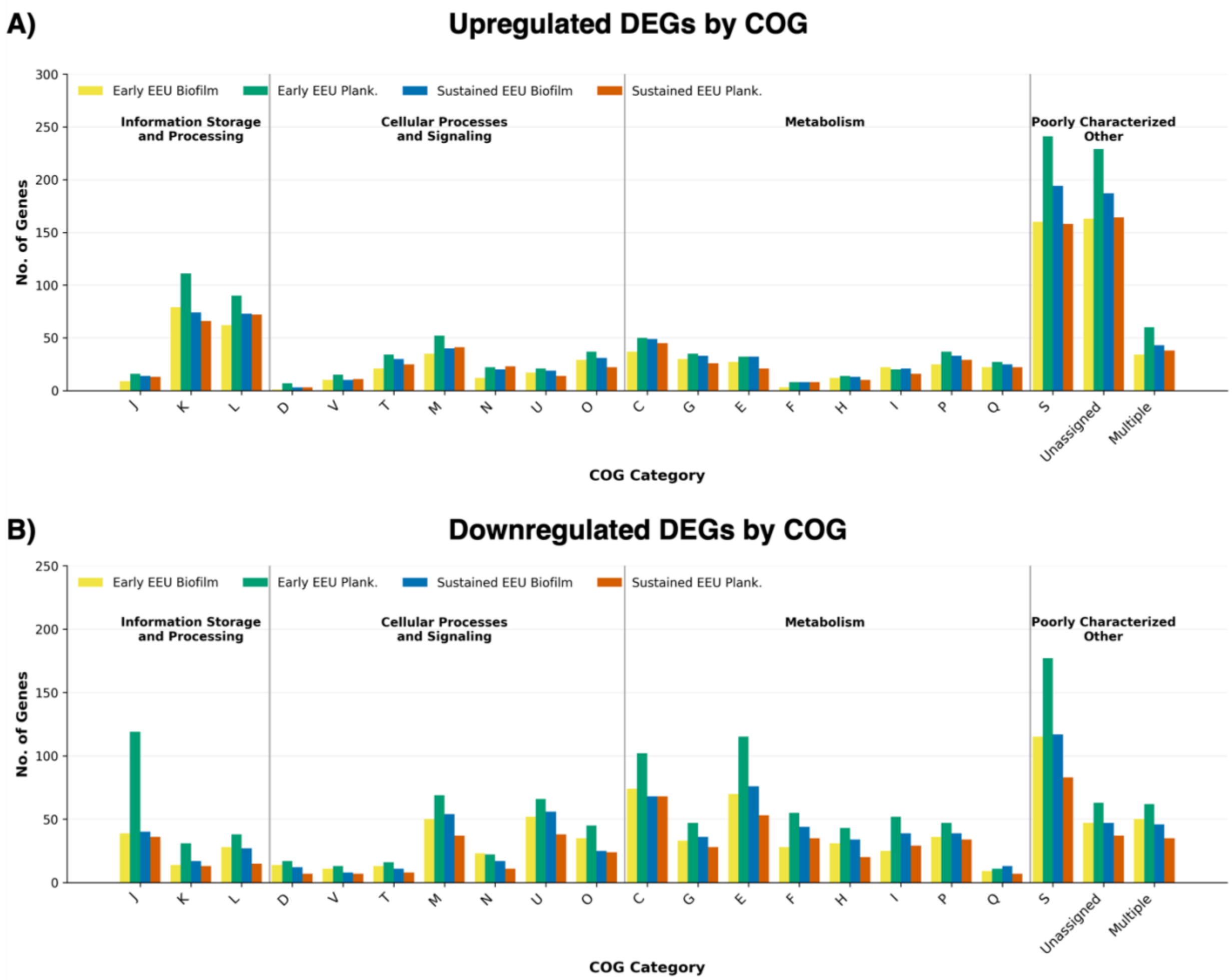

Figure S3. Distribution of differentially expressed genes (DEGs) across cluster of orthologous group (COG) functional categories for cathode oxidation samples. A) Number of upregulated per COG category relative to pre-growth control ($log_2FoldChange \geq 1$, $p_{adj} \leq 0.01$) . **B)** Number of downregulated genes per COG category relative to pre-growth control ($log_2FoldChange \leq -1$, $p_{adj} \leq 0.01$). Early EEU biofilm – yellow, early EEU planktonic fraction – green, sustained EEU biofilm – blue, sustained EEU planktonic fraction – orange. COG categories are grouped by function: Information Storage and Processing (J-L), Cellular Processes and Signaling (D-O), Metabolism (C-Q), and Poorly Characterized/Other (S, Unassigned, Multiple). Single-letter codes follow standard COG nomenclature. Early EEU planktonic samples show the highest number of DEGs across most categories.

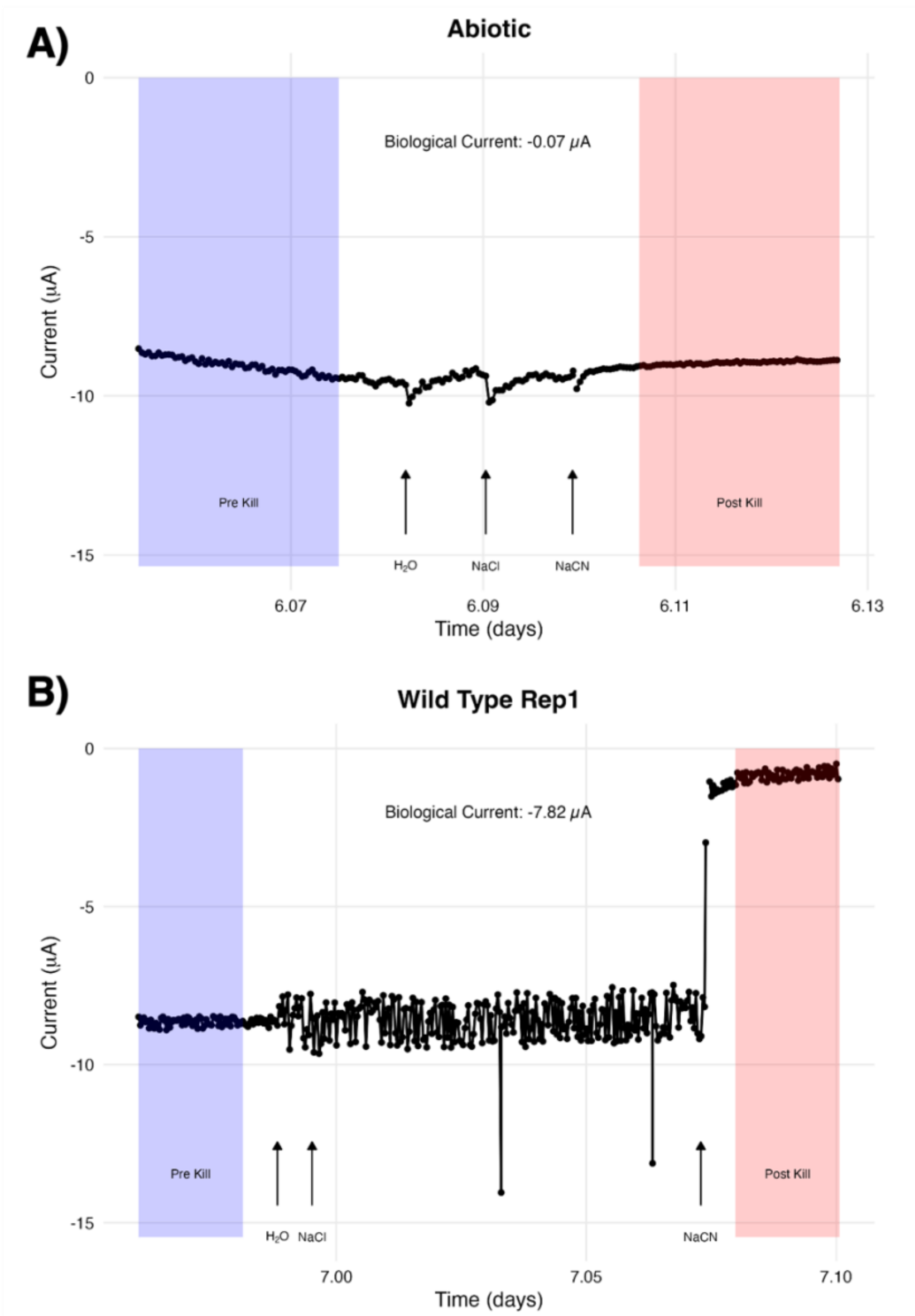

Figure S4. Representative biological current determination. (A) Abiotic control. **B)** Wild type *Thioclava electrotropha* biofilm. Water and 1M NaCl additions (200 µL each into reactors containing 20 mL of catholyte, indicated by arrows) were preformed to evaluate if nonfaradaic (capacitive) processes were contributing to current consumption at the electrode surface, of which none were observed for either the abiotic control or biological replicates. Upon addition of 200 µL of 1M NaCN, we observed no change in current in the abiotic control but measured a sharp decrease in current consumption by *T. electrotropha*. The difference in cathodic current pre additions and post kill (highlighted in blue and red, respectively), corresponds to biological current.

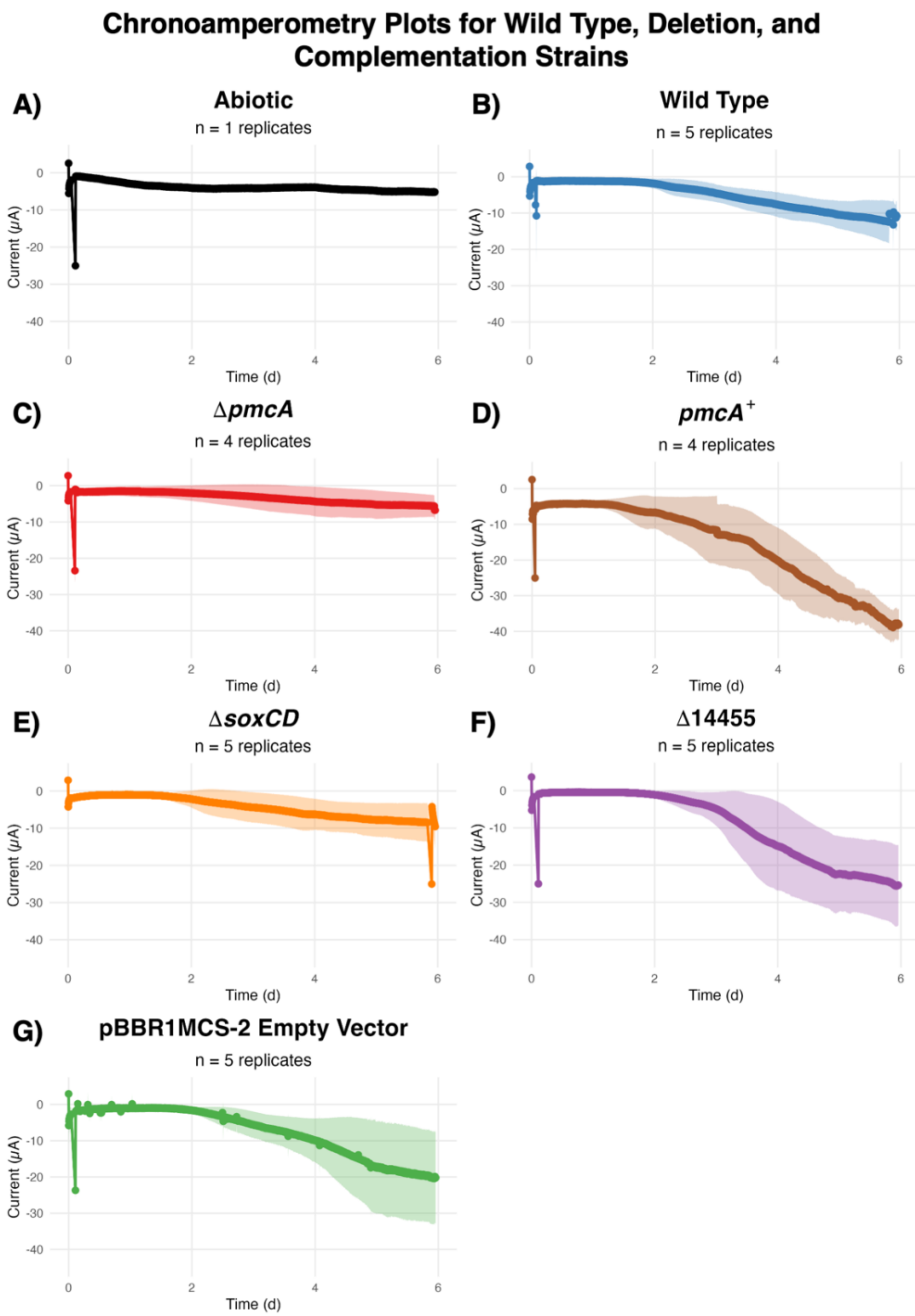

Figure S5. Chronoamperometry plots of wild type, deletion, and complementation strains of *Thioclava electrotropha*. Current consumption (mean ± 1 standard deviation) over ~6 days is plotted for all strains. Strain names are indicated in the title of each plot, with the number of replicates noted just below the title. Axes are identical among all plots, permitting direct comparisons. **A)** Abiotic control, 1 replicate. **B)** Wild type *Thioclava electrotropha*, 5 replicates. **C)** Δ*pmcA*, 4 replicates. **D)** *pmcA*$^{+}$, 4 replicates. **E)** Δ*soxCD*, 5 replicates. **F)** ΔAKL02_14455, 5 replicates. **G)** pBBR1MCS-2 empty vector control, 5 replicates.
